# Comparative genomic analysis reveals distinct genotypic features of the emerging pathogen *Haemophilus influenzae* type f

**DOI:** 10.1186/1471-2164-15-38

**Published:** 2014-01-18

**Authors:** Yu-Ching Su, Fredrik Resman, Franziska Hörhold, Kristian Riesbeck

**Affiliations:** Medical Microbiology, Department of Laboratory Medicine Malmö, Lund University, Jan Waldenströms gata 59, SE-205 02 Malmö, Sweden; Institute of Computer Science, Department of Mathematics and Computer Science, Friedrich-Schiller-University of Jena, PF 07737 Jena, Germany

**Keywords:** Comparative genomics, *Haemophilus influenzae* serotype f, Invasive, Pathogen, Synteny, Virulence factor

## Abstract

**Background:**

The incidence of invasive disease caused by encapsulated *Haemophilus influenzae* type f (Hif) has increased in the post-*H. influenzae* type b (Hib) vaccine era. We previously annotated the first complete Hif genome from a clinical isolate (KR494) that caused septic shock and necrotizing myositis. Here, the full genome of Hif KR494 was compared to sequenced reference strains Hib 10810, capsule type d (Hid) Rd Kw20, and finally nontypeable *H. influenzae* 3655. The goal was to identify possible genomic characteristics that may shed light upon the pathogenesis of Hif.

**Results:**

The Hif KR494 genome exhibited large regions of synteny with other *H. influenzae*, but also distinct genome rearrangements. A predicted Hif core genome of 1390 genes was shared with the reference strains, and 6 unique genomic regions comprising half of the 191 unique coding sequences were revealed. The majority of these regions were inserted genetic fragments, most likely derived from the closely-related *Haemophilus* spp. including *H. aegyptius, H. haemolyticus* and *H. parainfluenzae*. Importantly, the KR494 genome possessed several putative virulence genes that were distinct from non-type f strains. These included the *sap*2 operon, *aef*3 fimbriae, and genes for kanamycin nucleotidyltranserase, iron-utilization proteins, and putative YadA-like trimeric autotransporters that may increase the bacterial virulence. Furthermore, Hif KR494 lacked a *his*ABCDEFGH operon for *de novo* histidine biosynthesis, *hmg* locus for lipooligosaccharide biosynthesis and biofilm formation, the *Haemophilus* antibiotic resistance island and a *Haemophilus* secondary molybdate transport system. We confirmed the histidine auxotrophy and kanamycin resistance in Hif by functional experiments. Moreover, the pattern of unique or missing genes of Hif KR494 was similar in 20 Hif clinical isolates obtained from different years and geographical areas. A cross-species comparison revealed that the Hif genome shared more characteristics with *H. aegyptius* than Hid and NTHi.

**Conclusions:**

The genomic comparative analyses facilitated identification of genotypic characteristics that may be related to the specific virulence of Hif. In relation to non-type f *H. influenzae* strains, the Hif genome contains differences in components involved in metabolism and survival that may contribute to its invasiveness.

**Electronic supplementary material:**

The online version of this article (doi:10.1186/1471-2164-15-38) contains supplementary material, which is available to authorized users.

## Background

*Haemophilus influenzae* is a Gram-negative coccobacillus that commonly dwells in the human upper respiratory tract. In addition to asymptomatic colonization, the species causes a wide spectrum of respiratory tract infections. *H. influenzae* is, for example, associated with acute otitis media in children, with sinusitis and pneumonia in adults as well as exacerbations in patients with chronic obstructive pulmonary disease (COPD). *H. influenzae* also occasionally causes invasive disease such as meningitis and septicemia [1, 2].

Isolates without a polysaccharide capsule are designated as nontypeable *H. influenzae* (NTHi), whereas encapsulated and hence typeable isolates are further divided into 6 serotypes designated a to f, depending on the capsular polysaccharide composition and antigenicity. NTHi is a commensal in the upper respiratory tract, and mainly causes upper respiratory tract infections. On the other hand, encapsulated strains, and most significantly capsule type b (Hib), are associated with systemic disease, and used to be a common cause of meningitis and epiglottitis in small children. However, the carriage and disease of Hib in developed countries has been greatly reduced since the 1990s due to the widespread use of Hib-specific vaccines [1]. Before introduction of the Hib vaccine, invasive non-Hib infections received little attention, being vastly outnumbered by severe Hib infections. However, epidemiological studies of invasive cases reported between 1989–2010 from North America and Europe indicate that invasive *H. influenzae* disease is now predominantly caused by NTHi and type f (Hif) [3–6]. Detailed analyses suggest that while NTHi acts as a true opportunist in systemic disease, Hif is also opportunistic (affecting frail individuals with underlying co-morbidities or predisposing conditions such as COPD, alcohol abuse, malignancy and diabetes), but often presents as a severe invasive disease in previously healthy and immunocompetent individuals [3, 7–10]. Importantly, more than half of the cases of invasive Hif infection presented in previously healthy individuals, and more than one third of these patients needed further treatment at intensive care units [3].

The genetic mechanism underlying the virulence of Hif is presently unknown, particularly when compared to Hib. Due to the increasing clinical significance of Hif, attempts to characterize established *H. influenzae* virulence factors including the capsule, lipooligosaccharide (LOS), *hif* fimbriae, the adhesin Hap as well as antibiotic and serum resistance have been performed in clinical Hif isolates [11–14]. With the objective to increase the current body of knowledge on Hif, we recently sequenced and annotated the complete genome of Hif KR494 [9, 15]. This clinical isolate caused necrotizing myositis and septic shock in a previously healthy 70-year old man. The Hif genome consists of 1856176 bp of chromosomal DNA, from which 1742 intact coding sequences (CDSs) were identified.

The primary objective of the present study was to use the *de novo* assembled genome to determine the genetic characteristics of Hif, focusing on differences compared with other sequenced *H. influenzae* genomes of varying serotypes and infection sites. The secondary objective was to study the genetic conservation of these features in different Hif clinical isolates, and possibly associate it with the phenotype. Our analyses enabled delineation of the accessory genome and revealed a plethora of Hif-specific genomic features including gene acquisition and gene loss that might play a critical role for virulence and host adaptation.

## Methods

### Bacterial strains

*H. influenzae* laboratory strains (*n* = 2) and clinical isolates (*n* = 21) used in the present study are listed in Table 1. Bacteria were grown on chocolate agar or in brain heart infusion (BHI) broth supplemented with NAD (Sigma-Aldrich, St. Louis, MO) and hemin (Merck, Darmstadt, Germany) (each at 10 μg/ml) at 37°C in a humid atmosphere containing 5% CO_2_.

**Table 1:** **Laboratory strains and clinical** ***Haemophilus influenzae*** **isolates used in the present study**

Strain	Serotype ^a^	MLST ^b^	Year of isolation	Isolation site ^c^	Underlying conditions ^c^	Clinical manifestation ^d^	Gender	Age	Geographical location of isolation ^e^	Reference
**Laboratory strains**										
MinnA	b	n/a	1979	CSF	n/a	Septic shock	n/a	Child	Minneapolis, USA	[16]
3655	Nontypeable	n/a	n/a	Middle ear	n/a	AOM	n/a	Child	Missouri, USA	[17]
**Clinical isolates**										
KR494	f	124	2008	Blood	Healthy	Septic shock	Male	70	Skåne	[9]
G19	f	124	2008	Blood	n/a	n/a	Male	57	Gothenburg	[3]
G20	f	124	2008	Blood	n/a	n/a	Male	4	Gothenburg	[3]
K238	f	124	2007	Blood	n/a	n/a	Female	63	Stockholm	[3]
L11	f	n/a	2006	Blood	MDS	Bacteremia/sepsis	Male	65	Skåne	[3]
L16	f	n/a	2007	CSF	Healthy	Bacteremia/sepsis	Male	74	Skåne	[3]
L21	f	124	2008	Blood	Healthy	Septic shock	Male	82	Skåne	[3]
L22	f	124	2008	Blood	Severe COPD	Bacteremia/sepsis	Male	81	Skåne	[3]
L24	f	124	2009	Blood	Healthy	Bacteremia/sepsis	Male	65	Skåne	[3]
L25	f	124	2009	Blood	Healthy	Septic shock	Male	51	Skåne	[3]
L29	f	124	2009	Blood	Healthy	Septic shock	Male	77	Skåne	[3]
L45	f	n/a	2005	Blood	Healthy	Septic shock	Male	51	Skåne	[3]
L50	f	124	2007	Blood	Healthy	Septic shock	Male	63	Skåne	[3]
L59	f	n/a	2007	Blood	Healthy	Severe sepsis	Male	71	Skåne	[3]
M1	f	124	2009	Blood	leucemia	Bacteremia/sepsis	Male	3	Skåne	[3]
M10	f	124	2008	Blood	Solid tumor	Bacteremia/sepsis	Female	77	Skåne	[3]
M14	f	124	2008	Blood	Solid tumor	Bacteremia/sepsis	Male	63	Skåne	[3]
M29	f	n/a	2006	CSF	Healthy	Septic shock	Male	0	Skåne	[3]
M54	f	n/a	1999	Blood	Healthy	Bacteremia/sepsis	Female	78	Skåne	[3]
S208	f	124	2008	CSF	n/a	n/a	Female	61	Stockholm	[3]
S229	f	124	2008	Blood	n/a	n/a	Female	88	Stockholm	[3]

^a^Isolates were characterized by standard bacteriological techniques as described [3]. Strain serotype was verified by PCR whereas *H. haemolyticus* was excluded by 16S rRNA typing [3].

^b^Multilocus sequence typing (MLST) was performed as previously described [18].

^c^COPD, chronic obstructive pulmonary disease; CSF, cerebrospinal fluid; MDS, myelodysplastic syndrome.

^d^AOM, acute otitis media.

^e^Clinical isolates were from three different geographical areas of Sweden: Stockholm, Gothenburg and Skåne spanning from the years 1999–2009, isolated from blood or cerebrospinal fluid (CSF), but the clinical presentation, which was known for 15 of the 20 cases of invasive disease, ranged from Septic shock and epiglottitis to mild disease [3].

n/a, data not available.

### Genome alignment and rearrangement

Full genome alignment and comparison of genomic rearrangement patterns between the Hif KR494 and reference strains (Table 2) was performed using the following programs: Mummer program [19], Artemis Comparison Tool (ACT) [20] or mVISTA [21] with BLASTn setting at a minimum identity of 95% and an expected threshold = 1e^-5^ unless otherwise indicated. For initial genome sequence pairwise alignment, a default setting value of 70% was used as the minimum percent conservation identity that must be maintained over the window size 11 for a region (> 50 bp) to be considered conserved. Thereafter, from the total identified conserved genomic blocks, a minimum of 95% of sequence identity was set to identify highly conserved regions (>50 bp). Genomic comparative maps were visualized using ACT, whereas Artemis [22] was used for data management. GenBank accession numbers of genomes used in the present study are listed in Table 2.

**Table 2:** **General genome features of Hif KR494 and the reference** ***H. influenzae*** **and** ***Haemophilus*** **spp. strains**

	***H. influenzae***				***H. aegyptius***	***H. haemolyticus***	***H. parainfluenzae***
**Strain**	KR494	10810	RD Kw20	3655	ATCC 11116	M21639	ATCC 33392
**Serotype**	f	b	d	nontypeable	**-**	**-**	**-**
**GenBank accession**	CP005967	NC_016809	NC_000907	AAZF01000000	AFBC00000000	AFQR01000000	AEWU01000000
**Length (bp)**	1856176	1981535	1830138	1878368	1915025	2328161	2110314
**Whole genome GC content (%)**	38.05	38.14	38.15	38.02	38.08	38.59	39.18
**Genes GC content (%)**	38.68	38.76	38.83	38.75	39.00	39.33	39.83
**Coding content (%)**	88.54	87.29	84.66	89.38	90.07	74.29	91.59
**Genes avg. length (bp)**	913	910.79	935.59	870.79	856	887.6	962.55
**Total CDS number**	1742	1903	1657	1929	2020	2224	2010
**Strain-specific CDS** ^**a**^	-	408	199	448	553	773	644
**Common CDS** ^**b**^	-	1495	1458	1481	1487	1451	1366
**Unique CDS in Hif KR494 as compared to the other strains** ^**c**^	-	247	284	261	255	292	376

^a^CDS number of each strain or species that was less conserved (protein sequence <85% similarity) or absent in the Hif KR494 genome.

^b^CDS number of each strain or species that was conserved and homologous to the Hif KR494 genome (protein sequence ≥85% similarity).

^c^Number of CDS in Hif KR494 that were less conserved or absent as compared to the reference genomes (protein sequence <85% similarity).

### Comparative analysis of gene content

To find unique and common genes in the Hif KR494 and reference strains/species, we performed extensive comparative analyses of open reading frames (ORFs) from whole genome sequences. We used the Mummer program in these analyses at window size 11. Briefly, total ORFs from KR494 and a selected reference genome, or of a reference genome pair, were analyzed with tBlastx at the setting of cutoff e-value ≤ 1e^-5^ and protein sequence similarity ≥85%. Finally, proteins with the best hits value from reciprocal blast were initially collected and grouped as (i) common CDSs shared between genomes, and (ii) CDSs unique to each genome. Results were formatted in Blast m8 tabular form. Thereafter, we used Perl scripts to further retrieve the accessory genome of Hif KR494 using different parameters, that is, genes absent from (i) all *H. influenzae* reference genomes used in the present study (Table 2), (ii) related *Haemophilis* spp. reference genomes (Table 2) or (iii) genes not found in all *H. influenzae* genome sequences available in the current databases. A similar approach was used to obtain common CDSs using the same parameters as outlined above. DNA plotter [23] and ACT were used for visualization of genomic features. In the present study, protein sequence homology (over the complete protein length) between Hif KR494 and reference strains was presented in percentage of similarity and identity.

### Histidine auxotrophic assay

A histidine-dependent growth assay of *H. influenzae* isolates was performed with some modifications [24]. Two different media were used in this assay; Herriot defined media [25] with or without L-histidine denoted as w-His and w/o-His, respectively. Briefly, bacterial colonies were washed and resuspended in 5 ml of medium w/o-His to a cell density of OD_600_ 0.5. Thereafter, 5 × 10^3^ colony forming units (CFU) were separately inoculated into 10 ml of medium w/o-His and medium w-His supplemented with L-histidine at four different concentrations (0.0001%-0.01%). Here, the optimal concentration of L-histidine was empirically titrated for Hif isolates since the original Herriot defined medium was designed for *H. influenzae* serotype d [25]. Cultures were incubated for 12 h at 160 rpm and 37°C in a humid atmosphere containing 5% CO_2_. Bacterial growth was measured by spectrophotometry at OD_600_. BHI broth supplemented with NAD and hemin was used as a control medium.

### PCR-based gene distribution studies

Genomic DNA was extracted with a GenElute Bacterial Genomic DNA Kit according to the manufacturer’s instruction (Sigma-Aldrich). Primers used in gene distribution studies are listed in supporting information (Additional file 1). Seventeen primer pairs (denoted as Hif_U1 to Hif_U17) were designed based upon the ORFs identified in the Hif KR494 genome sequence and used to screen unique genes/operons of the Hif accessory genome. The remaining primer pairs (Hif_M1 to Hif_M11) were used to study the missing genes and were designed based on the conserved ORFs from Hid Rd Kw20 unless otherwise indicated. PCR reactions were performed according to a standard protocol and conditions were as indicated in Additional file 1.

### Antimicrobial susceptibility testing

Bacterial colonies were resuspended in sterile 0.9% NaCl to a density of McFarland standard 0.5. Antimicrobial susceptibility tests were performed using Etest® kanamycin strips (0.016-256 μg/ml) according to the manufacturer’s instruction (bioMérieux, Marcy l’Etoile, France). Minimum inhibitory concentration (MIC) of antibiotics was defined as the lowest concentration which fully inhibited the bacterial growth in comparison to antibiotic-free media.

## Results

### General features of the Hif KR494 genome

We first analyzed the KR494 genome based on a comparison with the well-established genomes of typeable and nontypeable *H. influenzae* that are available in the public databases. An overview of the complete genomes of Hif KR494 and selected reference strains are presented in Table 2. Genomes of NTHi are widely studied, and sequences from 18 distinct full NTHi genomes are available [26]. In contrast, only three annotated full genomes of typeable strains were available prior to this study, that is, Hib 10810, Hid Rd Kw20, and the recently reported Hif KR494 [15, 27, 28]. Due to their well-studied virulence characteristics and clinical significance, typeable strains of Hib 10810 and Hid Rd Kw20 in addition to NTHi 3655 were selected as references in our study. Regardless of the capsular serotype, the overall features of Hif KR494 were relatively similar to Hib 10810, Hid Rd Kw20 and NTHi 3655 (Table 2). The Hif KR494 genome was approximately 1.4% larger than the avirulent Rd Kw20, but 6.3% smaller as compared to Hib 10810. However, the Hif KR494 genome was smaller than the NTHi 3655 genome, but was relatively similar in size when compared to all NTHi genome sequences available (~1.8-1.95 Mb) [26, 29]. A similar G + C content (whole genome) of approximately ~38% was observed for the various serotypes (38.02-38.15%), whereas the average gene length varied only slightly between the different *H. influenzae* serotypes*.* In contrast, the numbers of predicted proteins and percentages of coding content in the four studied *H. influenzae* strains did not correlate with genome sizes and varied considerably. This could be due to the different annotation methods used for each genome. Notably, Rd Kw20 possessed the lowest number of CDSs, with the shortest genome among all analyzed.

### A comparison of the genome organization between Hif KR494 and other *H. influenzae*strains

We further analyzed the whole genome sequence similarity between Hif KR494 and the reference strains Hib 10810, Rd Kw20 and NTHi 3655. Due to genome rearrangement, whole genome DNA sequence similarity between strains appeared as a “block of conserved sequences” as analyzed by mVISTA. For this analysis, blocks were defined as contiguous regions of 50 bp to 100 kbp displaying a minimum of 70% nucleotide identity. These blocks were separated by genetic regions with lower levels of identity that could be of variable lengths in the aligned sequences. The Hif KR494 genome was aligned in 122 and 111 genomic blocks (comprising a sum of 774852 bp and 768482 bp, respectively, with at least 95% nucleotide identity) to the Hib 10810 and Hid Rd Kw20 genomes, respectively. The number of homologous blocks was reduced to 69 when Hif was aligned with the NTHi 3655 genome (a total of 494192 bp alignments with 95% identity). The data thus showed a high sequence similarity between the genome of Hif KR494 and the reference strains Hib 10810 and Hid Rd Kw20, whereas the Hif KR494 genome was slightly divergent from NTHi 3655.

**Figure 1:**
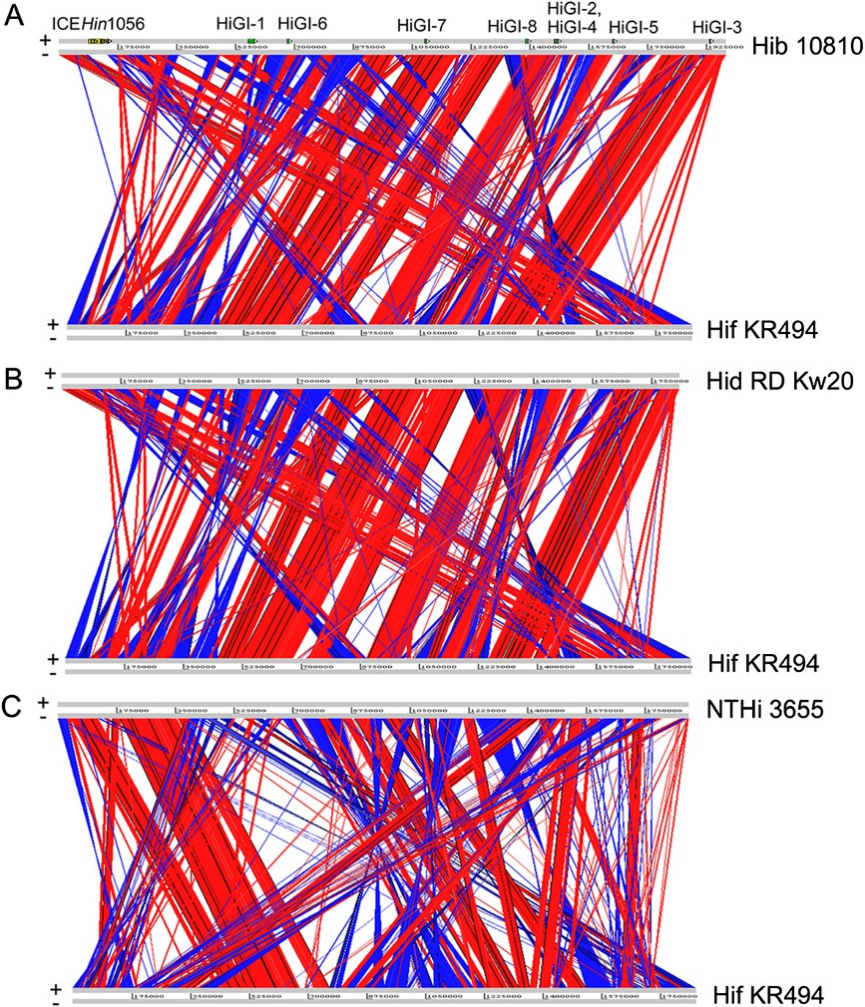
**Genome comparison of** ***H. influenzae*** **type f KR494 and reference strains in ACT view.** Genome alignment of Hif KR494 and **(A)** *H. influenzae* type b (Hib) 10810, **(B)** type d (Hid) Rd Kw20, and **(C)** nontypeable *H. influenzae* (NTHi) 3655. Respective genome designations are indicated on the right hand side of each genome line. Forward (+) and complement (−) strands of each genome are indicated in gray genome lines. Genomes are shown in full length and drawn to scale. Direct and inverted synteny between individual ORF (not indicated here) of the comparing genomes are shown in red and blue, respectively. The level of amino acid similarity is represented by colour shading with ascending saturation and indicates higher similarity. Genetic islands (HiGi) and the ICE element identified from Hib strain Eagan and strain 1056, respectively, are indicated for Hib 10810 in the upper genome line in panel **(A)**.

An alignment using the ACT was done to identify inserted or deleted regions in the KR494 genome relative to the reference strains. Results showed large regions of synteny (genomic gene order) between Hif and these strains (Figure 1). Extensive synteny was observed between Hib 10810 and Hid Rd Kw20, which were distinct from Hif KR494 and NTHi 3655 (Additional file 2), indicating a closer relationship between Hib and Hid as compared with other types analyzed [18, 30]. Despite the gene organization and synteny of the Hif genome suggested a closer genetic relationship to the capsulated reference strains than to NTHi, numerous gene rearrangements were evident compared with all *H. influenzae* reference strains. Notably, multiple inversions were identified in the Hif KR494 genome, and were concentrated to three distinct regions; the 5′ end (within a ~450 kb fragment), the central region (within nucleotide positions 797–889 kb and 993–1254 kb), and finally at the 3′ end (~419 kb fragment).

**Figure 2:**
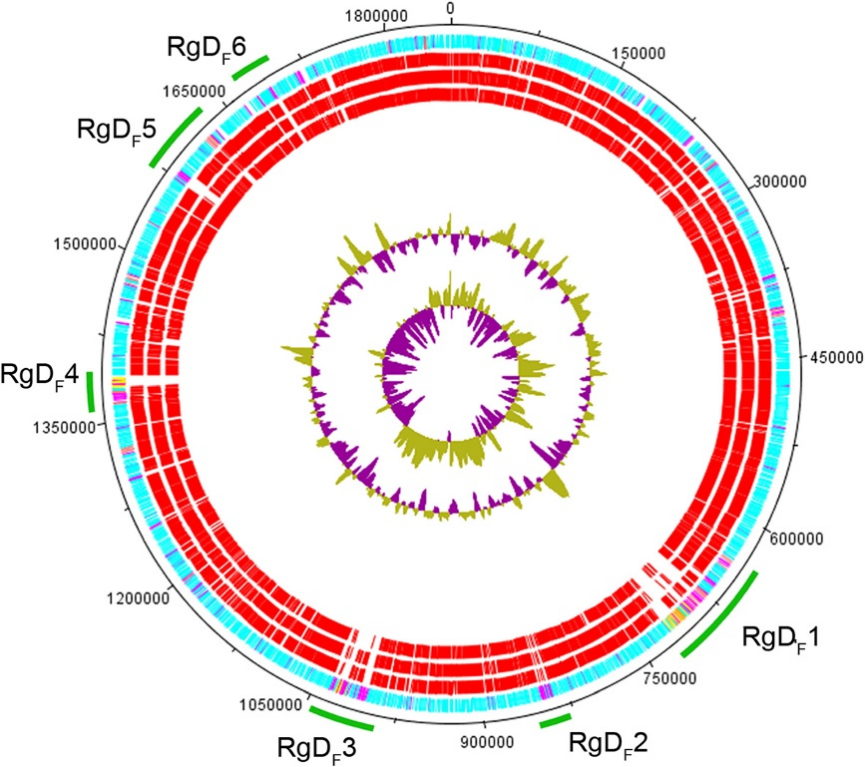
**Map of region of differences (RgD**_**F**_ **) of the** ***H. influenzae*** **type f KR494 genome.** Circular representation of protein conservation between Hif KR494 and reference strains was visualized using DNA plotter. From the outside in, the outer circle shows the genome length of Hif KR494 with position markers. The second circle shows the total ORFs of KR494 genome predicted on both forward and reverse strands. Common and unique ORFs relative to the reference strains are colored in blue and magenta, respectively. Phage-related ORFs are marked in yellow and orange. The third to fifth circles represent the distribution of individual ORF with high homology (≥85% similarity) (in red) to the corresponding ORF of reference strains, Hib 10810, Hid Rd Kw20 and NTHi 3655, respectively. Gaps between the conserved ORFs represent region of difference in the Hif KR494 genome, and were denoted as RgD_F_1 to 6 (marked with green lines). The GC plot and GC skew of the Hif KR494 genome are shown in the sixth and seventh circle, respectively.

In the Hif KR494 genome, 6 unique regions of difference (RgD) (*i.e.* relative to the reference strains) were identified and hereafter referred to as RgD_F_. The RgD_F_s were defined as regions in the Hif genome with a low level of conservation at the protein level relative to the reference strains (Hib 10810, Rd Kw20 and NTHi 3655), and containing a minimum of 5 neighboring CDSs with <85% sequence similarity. The RgD_F_s comprised a total of 144893 bp or 7.8% of the full genome (Figure 2). With the exception of RgD_F_3 and 6, the G + C contents of the remaining RgD_F_s (range 35.03-39.28%) were distinct from the remaining part of the genome (38.05%) (Additional file 3). This clearly indicated that the RgD_F_s were acquired from foreign source(s). The RgD_F_s contained several traits common to mobile genetic elements, including tRNA and rRNA genes, integrases (catalyze unidirectional DNA recombination), and transposases (catalyze movement of transposons). The majority of phage-related genes were found at RgD_F_1, 3 and 4, which were considered as prophage islands. The RgD_F_1, 4, 5 and 6 included the predicted Hif KR494 genomic islands (Gif_KR494_) 11, 13, 16 and 21, respectively (Additional file 4).

### The Hif KR494 accessory genome

**Figure 3:**
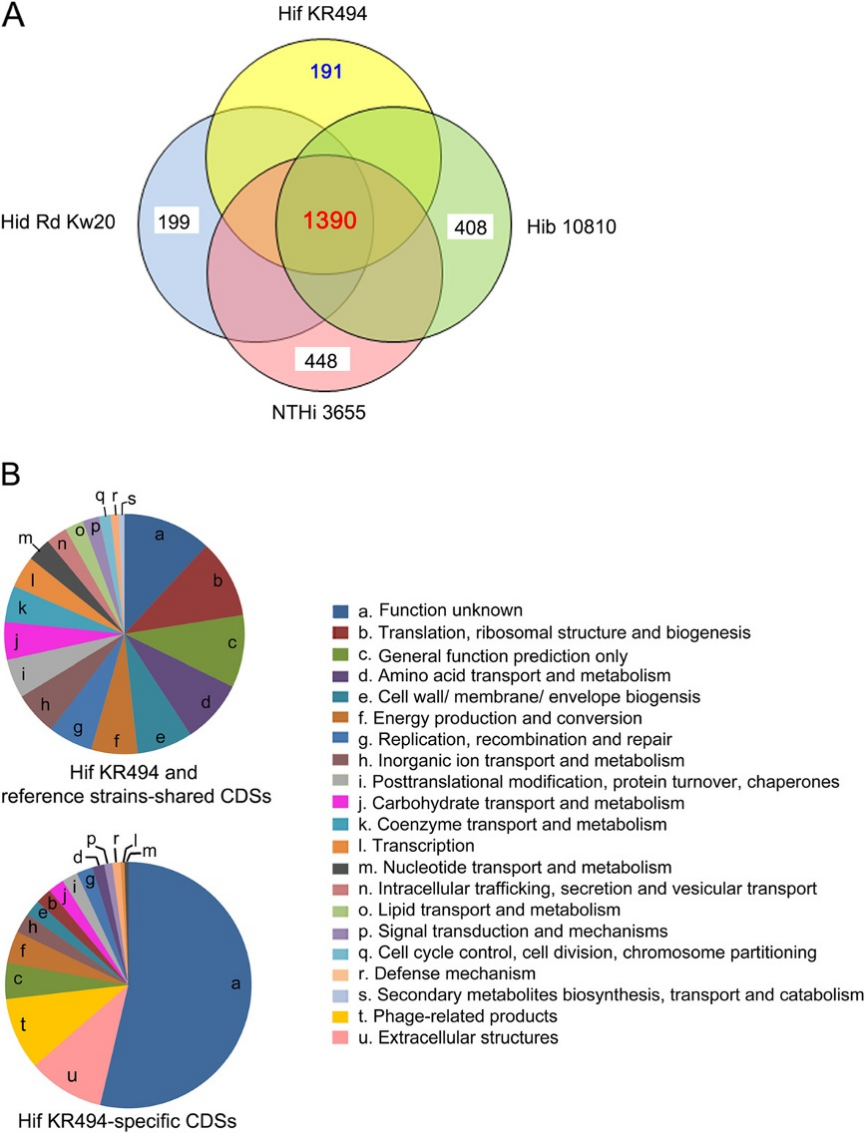
**Comparative genomic overview of** ***H. influenzae*** **type f KR494 and reference strains.** **(A)** A VENN diagrame depicts the number of commonly shared and strain-specific CDSs by Hif KR494 and reference strains. The total number of CDSs that are specific to Hif KR494 and conserved in all strains are shown in blue and red fonts, respectively. The number of strain-specific CDSs in Hib 10810, Hid RD Kw20 and NTHi 3655 compared to Hif KR494 are shown in black font. **(B)** Functional classification of subsets of KR494 CDSs shown in Panel **(A)**. Delineation was based on the COG database.

A pairwise BlastP comparison revealed 1390 CDSs of Hif KR494 shared with Hib 10810, Hid Rd Kw20 and NTHi 3655 when 85% similarity was used as cutoff [31, 32] (Figure 3A). The conserved CDSs included the *H. influenzae*-specific genes that were previously delineated as the *H. influenzae* core-genome (*i.e.* found in every strain) [26]. The distribution of gene functionality was analyzed based upon the Cluster of Orthologous Groups (COGs) protein database [33]. Besides the CDSs of unknown function, the majority of core genes were involved in protein translation, amino acid metabolism and cell wall biogenesis (Figure 3B). Since the *H. influenzae* core genome has been widely studied [26, 31, 34–37], the Hif counterpart was not further analyzed in the present study. Our analyses also showed that 408 CDSs were unique to Hib 10810, 199 CDSs were unique to Hid Rd Kw20, and finally 448 CDSs were unique to NTHi 3655 when compared to the Hif KR494 genome (Figure 3A).

Although Hif and the reference strains shared many homologous proteins, a detailed comparison revealed that 11% (191 CDSs) of the total annotated genes in the Hif KR494 genome were less conserved or absent from the reference genomes. These genes were thus further referred to as the Hif unique CDSs or accessory genes (Figure 3A, Table 3), which means that they were found in Hif KR494 only, but absent in the reference strains Hib10810, RdKw20 and NTHi3655. The distribution of accessory genes in the Hif KR494 genome verified the findings obtained in studies on the *H. influenzae* supragenome. Two previous supragenome studies revealed that 10-19% of the gene content in any *H. influenzae* genome is generally related to strain-specific accessory genes [26, 35]. As shown in Figure 3B, with the exception of products of unknown function, a significant number of the unique CDSs were associated with extracellular structures, *i.e.*, fimbriae and trimeric autotransporters. We also identified a number of unique CDSs encoding phage-related products. However, among all unique CDSs of Hif KR494, the majority (65.5%) showed a low homology or no significant hits in the *H. influenzae* genomes (summarized in Table 3).

**Table 3:** **Unique genes in Hif KR494 in relative to Hib 10810, Rd Kw20 and NTHi 3655**

Locus ^a^	Function	G + C ^b^ (%)	Homologue species	Similarity/Identity ^c^ (%)	USS ^d^	Mobile element ^e^	RgD _F_ /Gif _KR494_
**Capsule**							
HifGL_000665- HifGL_000673	Serotype-f capsule biosynthesis locus	25.5	*H. influenzae* 700222 (type f)	100/99.0-100	-	-	RgD_F_1
**Iron transport and metabolism**							
HifGL_000676- HifGL_000685*	*sap*2ABCDF	38.3	*H. parainfluenzae* ATCC 33392	94.0-100/87.0-96.0	4	Two class-LINE transposons (overlap with *sap*2C and transposase HifGL_000684); two transposase (HifGL_000683, HifGL_000684); phage island at downstream	RgD_F_1
HifGL_001444*	Iron Fe^3+^ ABC superfamily ATP binding cassette transporter	39.7	*H. haemolyticus* M21639	98.0/98.0	3	A class LINE/CR1 Transposon	-
HifGL_001664*	Heme-binding protein HutZ	38.5	*P. multocida* HN06	98.0/99.0	-	-	-
**Motility**							
HifGL_000989- HifGL_000994*	Fimbriae *aef*3abcdef	39.5	*H. aegyptius* ATCC 11116	62.0/52.0 (*aef*3a), 89.0-99.0/84.0-99.0 (*aef*3bcdef)	4	Flanked by two class LTR-Gypsy transposon (overlaps HifGL_000986) and transposase (HifGL_000996); phage island at downstream	RgD_F_3
**Energy production and conversion**							
HifGL_001349- HifGL_001358*	Rnf electron transport complex (*soh*B-*rnf*ABD)	39.8	*H. haemolyticus* M19501	91.0-100/85.0-100.0	12	Two identical direct repeats (41 bp) at HifGL_001351 and HifGL_001357; phage island at downstream	RgD_F_4
**Glycerol transport and metabolism of glycerophospholipid** ^**f**^							
HifGL_000396- HifGL_000397 (subunit gene duplication in *glp*KFQT operon)	Glycerol kinase GlpK	41.6	*H. influenzae* PittGG^f^	99.0/99.0	-	-	-
	Glycerol facilitator protein GlpF	43.6	*H. influenzae* PittGG^f^	99.0/99.0	-	-	-
**Posttranslational modification, protein turnover, chaperones** ^**f**^							
HifGL_000630-HifGL_000632 (duplication of three-gene cluster)	Phosphoethanolamine transferase	31.6	*H. influenzae* NT127^f^	99.0/99.0	-	-	-
	tRNA modification GTPase TrmE	39.7	*H. influenzae* NT127^f^	99.0/97.0	-	-	-
	Peptidylprolyl isomerase	34.3	*H. influenzae* R3021^f^	99.0/99.0	-	-	-
**Sugar and amino acid transport and metabolism** ^**f**^							
HifGL_000838- HifGL_000849 (a duplication of five-genes cluster)	Methyltransferase	37.4	*H. influenzae* R2866^f^	100/99.0	8	-	RgD_F_2
	Glycerol-3-phosphate regulon repressor GlpR	29.3	*Mannheimia succiniciproducens* MBEL55E	83.0/60.0			
	Glycerol-3-phosphate regulon repressor GlpR	33.2	*Aggregatibacter actinomycetemcomitans* D11S-1	93.0/85.0			
	Putative carbohydrate diacid regulator	37.1	*H. influenzae* R3021^f^	99.0/99.0			
	Gluconate proton symporter	44.1	*H. influenzae* NT127^f^	100/100			
**Kanamycin resistance (KNTase protein)**							
HifGL_000799*	DNA polymerase beta domain protein region	32.4	*M. haemolytica* PHL213	77.0/63.0	-	-	-
HifGL_000800*	Nucleotidyltransferase substrate binding protein	30.7	*M. haemolytica* PHL213	81.0/64.0	-	-	-
**Replication, recombination and repair**							
HifGL000956	DNA-damage-inducible protein J, negative regulator of translation	35.1	*H. influenzae* biogroup *aegytptius* F3031 (BPF)	98.0/97.0	-	-	-
HifGL001635*	Type II restriction enzyme HinfI	27.4	*H. influenzae* Rf (type f)	100/100	-	-	RgD_F_6
HifGL001636*	Adenine-specific methyltransferase HinfI	32.5	*H. influenzae* Rf (type f)	100/100	-	-	RgD_F_6
**Unknown function hydrolase**							
HifGL_000176*;	Cell wall-associated hydrolase	~48.0-49.0	*H. haemolyticus* M21639	100/100	-	Three transposons LINE/R2, LINE/R1 and LTR/Copia	Gif_KR494_-2
HifGL_000233;							Gif_KR494_-8
HifGL_001417;							Gif_KR494_-14
HifGL_001593;							Gif_KR494_-17
HifGL_001616;							Gif_KR494_-19
HifGL_001665							Gif_KR494_-22
**Trimeric autotransporter of unknown function**							
HifGL_000054	Putative YadA-like protein	43.3	*H. haemolyticus* M19107	83.0/ 77.0	-	-	-
HifGL_000835	Hep_Hag family protein	43.5	*Neisseria mucosa* ATCC 25996	63.0/ 45.0	-	-	-
HifGL_000837*	Putative target SNARE coiled-coil domain-containing protein	37.9	*H. haemolyticus* M19501	83.0/ 75.0	1	-	-
HifGL_000844	Putative YadA-like protein	40.3	*H. haemolyticus* M19501	99.0/ 99.0	1	-	-
HifGL_001158	Hep_Hag superfamily protein	32.9	*H. aegyptius* ATCC 11116	97.0/ 85.0	-	-	-
HifGL_001217	Trimeric autotransporter adhesin	37.4	*H. influenzae* biogroup *aegytptius* F3031 (BPF)	86.0/ 83.0	-	-	-
HifGL_001431*	Trimeric autotransporter adhesin	40.7	*H. influenzae* biogroup *aegytptius* F3047	86.0/ 80.0	1	-	-
HifGL_001626	YadA/hemagluttinin like protein	40.8	*H. aegyptius* ATCC 11116	88.0/84.0	-	-	RgD_F_6
**Other metabolic genes**							
HifGL000310*	Glutathione S-transferase domain-containing protein	40.8	*Actinobacillus ureae* ATCC 25976	98.0/99.0	-	-	-
HifGL000957	Addiction module antitoxin/rele toxin-like protein, plasmid stabilization system	33.3	*H. haemolyticus* M21127	100/98.0	-	-	-
HifGL001004	Phospholipase/carboxylesterase	33.1	*A. succinogenes* 130Z	89.0/79.0	-	-	-
HifGL001672*	Putative lipoprotein	33.8	*H. haemolyticus* M19501	95.0/93.0	-	-	-

^a^Unique CDS of hypothetical proteins and phage products are not included.

^b^Average G + C content is presented for single locus or as an average value of the indicated loci cluster or operon.

^c^Protein sequence similarity and identity (over the complete protein length) is presented in percentage as individual value for single CDS or as range of value for clustered CDSs.

^d^ *Haemophilus influenzae* uptake signal sequence (USS) (AAGTGCGGT).

^e^Mobile elements include phage island, transposase, tandem repeats and transposons. Transposon sequences and transposase, respectively, were predicted based on transposon database using softwares of RepeatMasker and RepeatProteinMask (http://www.repeatmasker.org/). Tandem repeat sequences were predicted by using Tandem Repeat Finder (http://tandem.bu.edu/trf/trf.html). Prophage islands were predicted using Prohinder software and ACLAME database (http://aclame.ulb.ac.be/), thereafter we performed BLAST comparison (standalone BLAT v.34) between KR494 genome and prophage database.

^f^These genes that exist in the Hif KR494 genome as a two-copy number resulted in unusual allelic duplication in the Hif KR494 genome. Despite these genes were considered unique in KR494 when compared to Hib 10810, Rd Kw20 and NTHi 3655, they also shared homology with NTHi PittGG, NT127, R3021 and R3866 that were not included as reference strains in the present study.

*Target single locus or contiguous loci assessed in gene distribution studies (Table 5).

### Putative virulence and metabolic genes unique to *H. influenzae*type f KR494

Approximately half of the total unique genes in Hif KR494 (114 CDSs) were located within the RgD_F_s. Notably, some of these unique CDSs resulted in duplication of paralogous genes (homologous genes present in the same strain, in this case Hif KR494) involved in virulence and iron utilization. In addition, allelic duplication was identified for genes involved in tRNA modification as well as in transport and metabolism of amino acids, sugar and glycerol (Table 3).

**Figure 4:**
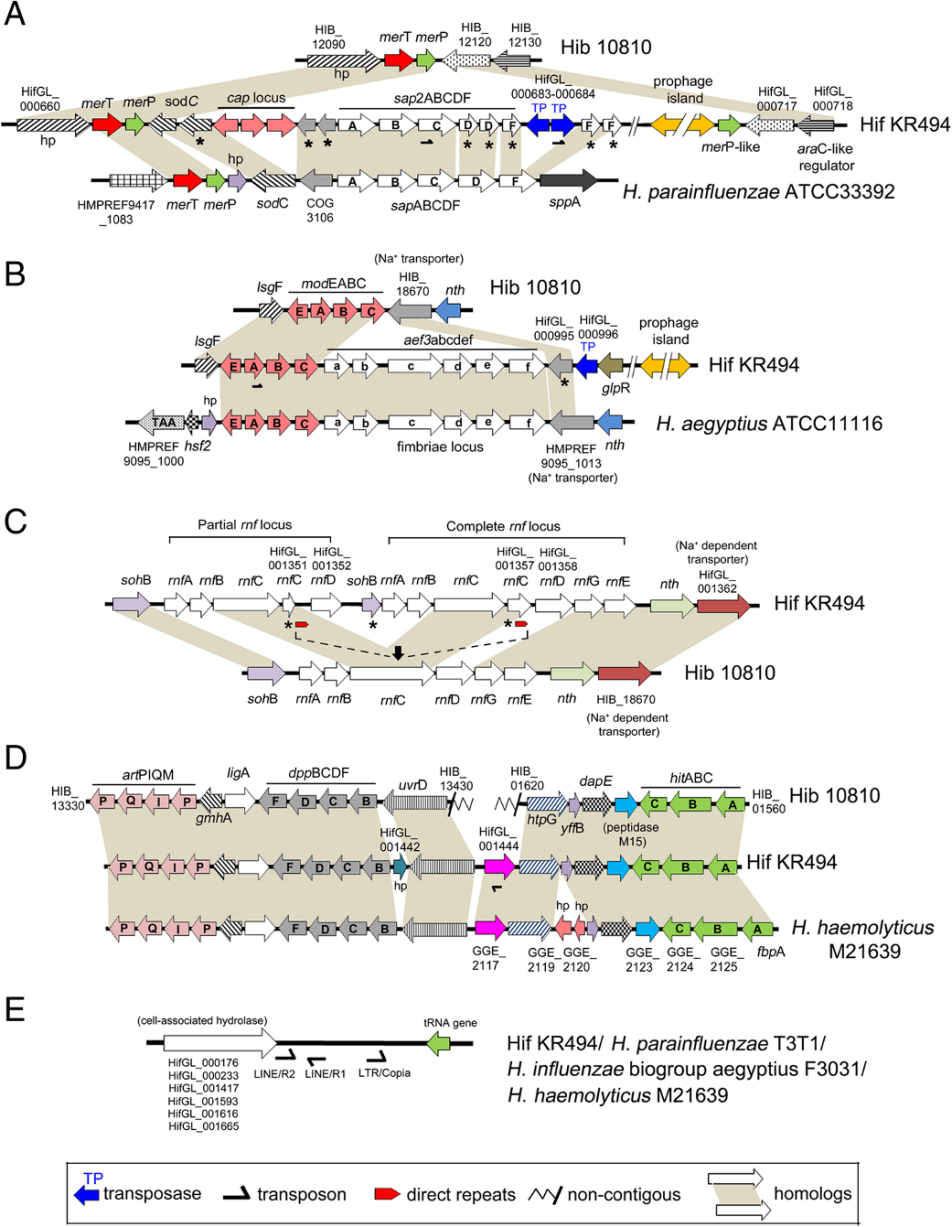
**Genomic structures and organizations of unique genes in** ***H. influenzae*** **type f KR494.** Organization of loci of specific genes in Hif KR494 were compared with reference strains or closely-related species. Genomes of respective reference species or strains are indicated on the right hand side of each panel. The flanking genes and genomic organization of **(A)** *sap*2ABCDF, **(B)** fimbriae gene cluster *aef*3abcdef, **(C)** duplet *rnf* electron transport complex, **(D)** unique iron-binding transporter HifGL_001444, and **(E)** genetic island structure of cell wall-associated hydrolase of KR494, were analogous to the indicated reference species while the unique genes were absent from Hib 10810 (a representative of *H. influenzae* reference genomes). Asterisks indicate partial CDSs. Hypothetical proteins of unknown function are denoted as “hp”. Homologous genes are indicated with gray shading. In panel **(A)**, the predicted protein products of *sap*2D and *sap*2F (ATPase subunits) are shorter than their counterparts in *H. parainfluenzae.* The loss of a functional ATPase complex (*sap*2DF) might be compensated by the subunit product (SapD and SapF) from the *H. influenzae* conserved Sap system. In panel **(C)**, two identical 41 bp direct repeats were identified at 40 bp upstream of HifGL_001352 (*rnf*D) and at the first 220 bp of HifGL_001357 (*rnf*C), respectively. The repeats may mark the two edges of the inserted genomic fragment suggesting the intergenic region between HifGL_001351 and HifGL_001358 as the insertion site. The black arrow indicates the possible insertion at the original *rnf*C subunit gene. The insertion may also have resulted in partial CDSs of the neighboring loci, HifGL_001351 and HifGL_001357. Both loci encode *rnf*C but with internal stop codon thus may not encode a functional protein. Nevertheless, the functionality of the *rnf* operon might not be affected since the intact CDS of *rnf*C were retained at HifGL_001350 and HifGL_001356.

The “*s*ensitivity to *a*ntimicrobial *p*eptide” (Sap) transporter of *H. influenzae* is a six-subunit multifunctional inner membrane ABC transport protein complex important for resistance against antimicrobial peptides (AMPs) [38, 39]. It consists of a periplasmic solute binding protein (subunit SapA), transmembrane permeases complex (SapB and SapC), ATPase subunits (SapD and SapF) and a subunit SapZ of unknown function. Two *sap* operons were identified in the Hif KR494 genome, a highly conserved *H. influenzae sap*ABCDFZ operon (HifGL_001309-HifGL_001314), and an additional five-gene *sap* operon encoded by unique Hif CDSs (HifGL_000676-HifGL_000685). The additional Sap operon shared a high protein sequence homology (94-100% similarity) with the SapABCDF operon of *H. parainfluenzae* ATCC33392 (corresponding locus in ATCC33392: HMPREF9417_1073 to HMPREF9417_1077), which is distinct from the conventional *sap* of *H. influenzae* (Table 3)*.* Moreover, the Hif KR494 additional *sap* operon lacked *sap*Z and its 5′ end coded for a COG3106 family hypothetical protein (HifGL_000675), and the entire gene organization was analogous to the *H. parainfluezae sap* operon (Figure 4A)*.* We thus annotated the unique operon in Hif KR494 as *sap*2ABCDF, and the numerical designation was to distinguish it from the “conventional” *sap* operon.

Interestingly, the well-studied Hif *cap* locus (HifGL_000665-HifGL_000673) was located a few CDSs upstream of the *sap*2 operon. The Hif KR494 *cap* locus was organized in a *sodC-cap* arrangement, a typical gene organization of group II capsule biosynthesis loci [13, 40]. A prophage island (HifGL_000691-HifGL000715) was located downstream of the *sap*2, and this island contained high numbers (15 CDSs) of *H. influenzae* and *H. aegyptius* phage protein homologues that were interspersed by hypothetical proteins (10 CDSs). The Hif capsule locus, *sap*2 and the prophage island together formed the RgD_F_1. The *sap*2 operon carried two class-LINE transposons (one overlapped with *sap*2C and one with transposase HifGL_000684) and two transposase genes (HifGL_000683 and HifGL_000684) that resembled a composite transposon-like structure (Figure 4A).

Fimbriae, also designated as hemagglutinin pili, are crucial for *H. influenzae* adherence and colonization in the upper respiratory tract [41, 42]. In addition to the classical *Haemophilus* fimbriae locus *hif*ACDE of genotype IIIb (HifGL_001282-HifGL_001285) that does not encode the periplasmic chaperon of subunit HifB [43], the Hif KR494 genome possessed another six-gene fimbriae cluster (HifGL_000989-HifGL_000994). The second fimbriae locus in Hif KR494 was found in the RgD_F_3, and had a high similarity (89-99% except for *aef*A = 62%) with the Aef fimbriae of *H. aegyptius* ATCC11116 (corresponding locus HMPREF9095_1007-HMPREF9095_1012) (Table 3). The *aef* homologue was not found in other *H. influenzae*, but is present as an *aef3*abcdef cluster in the conjunctivitis strain *H. influenzae* biogroup *aegyptius* F3047 (corresponding locus HICON_14070-HICON_14120) [29, 44]. We annotated the second Hif KR494 fimbriae cluster as *aef*3abcdef. While the *hif* cluster was inserted at the conserved region between *pur*E and *pep*N in the Hif KR494 chromosome, the *aef*3 cluster was located at a unique position between the *mod*C gene (HifGL_000988) and the sodium dependent transporter gene (HifGL_000995), analogous to the gene order found in *H. aegyptius* and *H. influenzae* biogroup *aegyptius* F3047 (Figure 4B). Two flanking mobile genetic elements were identified; a class LTR-Gypsy transposon (overlapped locus *mod*A; HifGL_000986) located upstream of the *aef*3 cluster, and a transposase IS1016C2 (HifGL_000996) located downstream of the same cluster, suggesting a composite-like structure of *aef*3. In addition, a small prophage island (HifGL_1021-HifGL_1026) was found a few CDSs downstream of the *aef*3 cluster.

NADH oxidoreductase is a six-subunit enzyme complex responsible for electron transfer to nitrogenase during nitrogen fixation [45]. The enzyme complex is encoded by a single copy *rnf*ABCDGE operon that is highly conserved in *H. influenzae*. However, the Hif KR494 genome had two contiguous *rnf* operons (HifGL_001348-HifGL_001360) consisting of a partial (*rnf*ABCD) and a complete operon (*rnf*ABCDGE) (Figure 4C). This may have been caused by insertion of an additional *rnf* gene cluster (HifGL_001352-HifGL_001357) via homologous integration between subunit genes of the original *rnf* operon. The suggested mechanism of gene insertion is illustrated in Figure 4C. Interestingly, the protein products of *rnf*A, *rnf*B and *rnf*D in Hif KR494 were homologous (98-100% similarity) to the corresponding Rnf proteins of *H. haemolyticus* (Table 3). Moreover, the third prophage island (HifGL_001362-HifGL_001379) that mainly encodes *H. haemolyticus* phage proteins was located downstream the Hif KR494 *rnf* operon. Both the *rnf* gene cluster and the adjacent prophage island were located on RgD_F_4.

RgD_F_6 contained unique CDS for the type-II restriction enzyme HinfI (HifGL_001635) and modification methylase HinfI (HifGL_001636) that were previously described in *H. influenzae* Rf (serotype f) [46]. The gene products are important for Hif to survive infection by a variety of phages. These genes have neither been identified in any non-type f *H. influenzae* genome nor characterized in previous genotyping studies of multiple Hif isolates [11, 12].

There were also a number of unique Hif CDSs that were not associated with the RgD_F_s (Table 3). These included two unique Hif CDSs (HifGL_000799 and HifGL_000800) that encoded hypothetical proteins containing nucleotidyltransferase and a substrate-binding domain (pfam08780 homologues of *Staphylococcus aureus* kanamycin nucleotidyltranserase (KNTase)), respectively. In *S. aureus,* the KNTase is a plasmid-encoded enzyme that confers resistance to a wide range of aminoglycoside antibiotics including kanamycin A [47, 48]. A BLAST analysis revealed that HifGL_000799 and HifGL_000800 are not present in other *H. influenzae* but have moderate homology with the hypothetical proteins MHA_2776 (77% similarity) and MHA_2775 (81% similarity) of *Mannheimia haemolytica* PHL213, respectively, and also share the direct synteny of flanking genes (illustration not shown)*.*

The Hif KR494 genome also harbored two genes involved in heme/iron utilization (HifGL_001444 and HifGL_001664) that have not been previously found in *H. influenzae*. The HifGL_001444 encodes a product of high homology (98% similarity) to the iron (Fe^3+^) ABC transporter substrate-binding protein of *H. haemolyticus* M21639 (locus in M21639: GGE_2117) (Table 3). It is distinct from the previously described heme/iron uptake systems in *H. influenzae*, *i.e.*, HxuABC, DppBCDF, hFbpABC, HbpA, Hgp, Hup, TbpAB, HipABC, P4 and Sap [38, 49–51]. Moreover, 11 upstream and 7 downstream flanking genes of HifGL_001444 had a similar gene order as in the *H. haemolyticus* M21639 genome (Figure 4D). The gene order included two relevant yet highly conserved *H. influenzae* heme/iron uptake systems, the *dpp*BCDF gene cluster (HifGL_001438-HifGL_001441) and the *hit*ABC operon (hFbpABC complex) (HifGL_001449-HifGL_001451). However, these were in different gene order as compared to the known *H. influenzae* reference genomes (Figure 4D). The HifGL_001664 encodes a heme-binding HutZ homologue (98% similarity) (GenBank number: YP_005362747) of *Pasteurella multocida*[52]. However, two typical gene partners of *hut*Z in a triplet gene operon, *hut*X and *hut*W of unknown function, were not present.

Another unique feature of the Hif KR494 genome was the presence of 6 copies of a CDS located on six ~3.0 kbp long genetic islands (Gif_KR494_-2, 8, 14, 17, 19 and 22) (Figure 4E, Table 3, Additional file 4). The CDS encodes a protein with high homology (100% similarity) to the cell-wall associated hydrolase (YP_004135972) of Brazilian purpuric fever (BPF) clone F3031 of *H. influenzae* biogroup *aegyptius*, *H. haemolyticus* M21639 (EGT78466) and *H. parainfluenzae* T3T1 (YP_004822531). No homologue of this protein has previously been found in other *H. influenzae*. Three transposons of class LINE/R2, LINE/R1 and LTR/Copia were consistently found at 41 bp, 336 bp and 1045 bp downstream of these CDSs, respectively. A tRNA gene was also present at ~2.5 kbp downstream of the CDSs. This implied that the cell-wall associated hydrolase has been horizontally acquired as a genetic island and subsequently integrated at the adjacent tRNA gene. Finally, additional genes encoding for a total of 7 unique YadA-like trimeric autotransporters with varying lengths (213-629aa) were also identified (Table 3). The exact function of these proteins is presently unknown.

### Gene deletions in the Hif KR494 genome

The Hif KR494 genome was also compared to the reference strains in order to define absent genes, that is, missing gene or gene loss in Hif KR494 but present in the reference strains Hib 10810, Rd Kw20 and NTHi 3655. Notably, when compared to Hib 10810, neither the previously described Hib genetic islands (HiGI-1 to HiGI-8 except for HiGI-6; originally identified from strain Hib Eagan) nor the integrative and conjugative element (ICE) *Hin1056* (strain Hib 1056) were present in the Hif KR494 genome (Figure 1A) [53–56]. The ICE*Hin*1056 is known to confer ampicillin, tetracycline and chloramphenicol resistance among *H. influenzae* transconjugants [54].

The Hif KR494 missing genes of non-hypothetical and non-phage proteins are summarized in Table 4. Unlike the majority of *H. influenzae* strains, the Hif KR494 genome did not retain the *his*ABCDEFGH operon (corresponding locus in Rd Kw20: HI0468-HI0474) and *his*IE (HI0475). The operon encodes 8 enzymes that co-operatively catalyze the formation of L-histidine from phosphorybosyl phyrophosphate, a crucial pathway in histidine biosynthesis [57]. Another important operon that was absent in the Hif KR494 was the eight-gene *hmg* locus (HI0867-HI0874) involved in LOS biosynthesis. The *hmg* locus is responsible for incorporation of sialyl- and (*P*Etn → 6)-α-D-Gal*ρ*NAc containing tetrasaccharide units which results in high molecular weight-glycoforms of LOS [58, 59].

**Table 4:** **Genes of reference** ***H. influenzae*** **strains that are absent in the Hif KR494 genome**

Locus ^a,b^	Gene/function
Histidine biosynthesis pathway	
HI0468-HI0474*	*his*ABCDEFGH operon
HI0475	*his*IE
Other amino acid biosynthesis pathway	
HI0607	Shikimate 5-dehydrogenase, aroE (phenylalanine, tyrosine and tryptophan)
HI0737	Acetohydroxy acid synthase II (valine, leucine and isoleucine)
Anaerobic fermentation of L-ascorbate	
HI1024-HI1026*	*ula*DEF operon
HI1027	*Lyx*
HI1031	2,3-diketo-L-gulonate reductase
Folate biosynthesis	
HI1190*	6-pyruvoyl tetrahydrobiopterin synthase
HI1464	Dihydropteroate synthase
Secondary molybdenum transporter	
HI1469	*mod*A
HI1470-HI1472*	*mol*ABC
HI1473-HI1475	*mod*D-*sal*X-*cys*U
HI1525	*mod*A
LOS biosynthesis	
HI0867-HI0874*	*hmg* locus (*sia*A, *wba*P, *rff*G)
HI1578	Glycosyl transferase WcaA
HIB_08850^c^	UDP-glucose--lipooligosaccharide glucosyltransferase
Iron and zinc transporter	
HIB_07090^c,^ *	Zinc transporter ZitB
HI0291	Periplasmic mercury transport-like protein
HI0661	Hemoglobin-binding protein OapA
HI0712	Hemoglobin-binding protein
Other metabolism	
HI0053	Zinc-type alcohol dehydrogenase (fructose and mannose metabolism)
HI0692	Xanthine-guanine phosphoribosyltransferase (purine metabolism)
HI1649	D-lactate dehydrogenase (pyruvate metabolism)
Others	
HI0584	Peptidase, aminobenzoyl-glutamate utilization protein
HI0947	Virulence-associated protein C
HI1251	Virulence-associated protein A
HI1465	Cell division FtsH-like protein

^a^CDSs of hypothetical proteins and phage products are not included.

^b^Locus tags are based on the genome of Rd Kw20 unless otherwise is indicated.

^c^Locus tags are based on the genome of Hib 10810.

*Target single locus or contiguous loci assessed gene distribution studies (Table 6).

Multiple molybdate transport systems of different affinities have previously been described in *H. influenzae* Rd Kw20 [60, 61]. In contrast to the high affinity *Haemophilus* primary molybdenum transporter operon *mod*ABCE that remained intact (HifGL_000985-HifGL_000988), the entire *mol*ABC-*mod*AD-*sal*X gene cluster (HI1469-HI1475) that encodes the secondary molybdenum transporter system was missing in the Hif KR494 genome. The gene for a ZitB zinc transporter (corresponding locus in Hib 10810: HIB_07090) was also absent in Hif KR494 genome. In parallel, the genes HI1024-HI1027 and HI1031 coding for enzymes involved in anaerobic fermentation of L-ascorbate as an alternative carbon source were not present in the Hif KR494 genome. Consistent with the previous description of Hif isolates [11, 12], the Hif KR494 genome did not harbor genes for the high molecular weight adhesin (*hmwAB*), which is a common virulence factor in NTHi.

### The unique genetic properties of Hif KR494 are conserved in clinical type f isolates

To investigate whether the distinct genomic features in the Hif KR494 genome were conserved in the serotype f lineage, we investigated the distribution of the unique and missing genes in 20 clinical Hif isolates using a PCR-based screening. The clinical isolates were chosen from different years and from various geographical areas of Sweden (Table 1). The severity of clinical disease had been established for most isolates used in the present study, and ranged from mild disease in immunocompromised individuals to septic shock in previously healthy subjects.

To avoid orthologs, (homologous genes present in heterologous *H. influenzae* strains), we targeted 17 unique CDSs that lack homology with the *H. influenzae* accessory gene pool available in the database (Table 5, Additional file 1). The well-studied NTHi 3655 and Hib MinnA (genetically clonal to Hib 10810) were used as negative controls, whereas Hif KR494 represented the positive control. Using primers based upon Hif KR494 sequences, 10 Hif isolates were positive for all the unique CDSs screened, indicating the presence of the targeted genes, whereas the remaining isolates were negative for one to a maximum of four genes (Table 5).

**Table 5:** **Distribution of Hif KR494 unique genes in clinical Hif isolates**

Locus tag ^a^ (HifGL)	Description ^b^	***H. influenzae*** serotype f clinical isolates																							
		KR494	G19	G20	K238	L11	L16	L21	L22	L24	L25	L29	L45	L50	L59	M1	M10	M14	M29	M54	S208	S229	No. of isolate ^c^	Hib MinnA	NTHi 3655
000176	cw-hydrolase	+	+	+	+	+	+	+	+	+	+	+	+	+	+	+	-	+	+	+	+	+	19	+^d^	+^d^
000310	GST	+	+	+	+	+	+	+	+	+	+	+	+	+	+	+	+	+	+	+	+	+	20	-	-
000674^a^	RgD_F_1: *sap*2	+	+	+	+	+	+	+	+	+	+	+	+	+	+	-	+	+	+	+	+	+	19	-	-
000799^a^	KNTase	+	+	+	+	+	+	+	+	+	+	+	+	+	+	+	+	+	+	+	+	+	20	-	-
000834^a^	RgD_F_ 2: *glp*R	+	+	+	+	+	+	+	+	+	+	+	+	+	-	+	+	+	+	+	+	+	19	-	-
000837	TAA	+	+	+	+	+	+	+	+	+	+	+	+	+	+	+	+	+	+	+	+	+	20	-	-
000989^a^	RgD_F_ 3: *aef*3	+	+	+	+	+	+	+	+	+	+	+	+	+	+	+	+	+	+	+	+	+	20	-	-
001007^a^	RgD_F_ 3:PI	+	+	+	+	+	+	+	+	+	+	+	+	+	+	-	+	+	+	-	+	+	18	-	-
001350^a^	RgD_F_ 4: *rnf*	+	+	+	+	+	+	+	+	+	+	+	+	+	-	+	-	+	+	+	+	+	18	-	-
001363^a^	RgD_F_ 4: PI	+	+	+	+	+	+	+	+	+	+	+	+	+	+^e^	-	+	+	+	+	+	+	19	-	-
001431	TAA	+	-	-	+	+	-	+	+	-	+	+	-	+	-	+	-	-	+	+	+	+	12	-	-
001444	Fe^3+^ transporter	+	+	+	+	+	+	+	+	+	+	+	+	+	+	+	+	+	+	+	+	+	20	-	-
001463	UHP	+	+	+	+	+	+	+	+	+	+	+	+	+	+	+	+	+	+	+	+	+	20	-	-
001635	RgD_F_ 6: RHinfI	+	+	-	+	+	+	+	+	+	+	+	+	+	+	+	+	+	+	+	+	+	19	-	-
001636	RgD_F_ 6: MHinfI	+	+	+	+	+	+	+	+	+	+	+	+	+	-	+	+	+	+	+	+	+	19	-	-
001664	HutZ	+	+	+	+	+	+	+	+	+	+	+	+	+	+	+	+	+	+	+	+	+	20	-	-
001672	Lpp	+	+	+	+	+	+	+	+	+	+	+	+	+	+	+	+	+	+	+	+	+	20	-	-
No. of CDS present		17	16	15	17	17	16	17	17	16	17	17	16	17	13	14	14	16	17	16	17	17		1	1

^a^Locus tags were based on the Hif KR494 genome [15]. For PCRs targeting the contiguous loci, only the first locus is shown. Full information of the target genes, amplicon size and PCR conditions is described in Additional file 1.

^b^Abbreviations: cw-hydrolase, cell wall-associated hydrolase; *glp*R, duplication of gene cluster involved in sugar and amino acid transport and metabolism; GST, glutathione S-transferase domain-containing protein; KNTase, kanamycin nucleotidyltranserase; Lpp,unknown lipoprotein; MHinfI,type f-specific adenine-specific methyltransferase HinfI; PI, prophage island; TAA, trimeric autotransporter; UHP, unknown function hypothetical protein; RHinfI,Type II restriction enzyme HinfI.

^c^Total number of Hif clinical isolates (excluding Hif KR494) with unique genes found in Hif KR494.

^d^CDS was not found despite the presence of primer priming site in the NTHi 3655 genome (AAZF01000000) and Hib 10810 (NC_016809); PCR product had a smaller size.

^e^Positive, but with a larger PCR product.

^+^PCR positive (CDS present); ^-^PCR negative (CDS absent).

In the screening of gene loss in Hif (in total 11 CDSs) (Table 6), the NTHi 3655 and Hib MinnA were used as positive controls. The LOS *hmg* locus (*sia*A and *wba*P) was lacking in all isolates analyzed, whereas 25% of the Hif isolates were positive for the gene encoding the ZitB transporter. Only two isolates carried the *mol*C gene, but were negative for *mol*A, indicating a partial deletion of the *mol* operon. In addition, all Hif isolates exhibited a full or partial deletion of the histidine biosynthesis operon. Our data thus suggested that the pattern of unique or missing genes in Hif KR494 was consistent in different Hif isolates. There was no specific genetic features that distinguished isolates from different geographical areas, or that could predict clinical severity. The latter observation also highlighted the importance of host factors in clinical disease.

**Table 6:** **Distribution of genes absent in Hif KR494 and other Hif clinical isolates**

Locus ^a^	Description/gene ^b^	Serotype f clinical isolates																							
		KR494	G19	G20	K238	L11	L16	L21	L22	L24	L25	L29	L45	L50	L59	M1	M10	M14	M29	M54	S208	S229	No. of isolate ^c^	Hib MinnA	NTHi 3655
Histidine biosynthesis																									
HI0468	*his*G	-	-	-	-	-	-	-	-	-	-	-	-	-	-	-	+	-	-	-	+	-	2	+	+
HI0469	*his*D	-	+	+	-	-	-	-	-	-	-	-	-	-	-	+	-	-	-	-	-	-	3	+	+
HI0470	*his*C	-	+	+	-	+	-	+	-	+	-	+	-	+	+	+	+	+	-	-	+	-	12	+	+
HI0472	*his*H	-	-	+	-	-	-	-	-	-	-	-	-	-	-	+	-	-	-	-	-	-	2	+	+
Anaerobic fermentation of L-ascorbate																									
HI1024	*ula*D	-	-	-	-	-	-	-	-	-	-	-	-	-	+	-	-	-	-	-	-	-	1	-^d^	-^d^
Folate biosynthesis																									
HI1190	PTS	-	-	-	-	-	-	-	-	-	-	-	-	-	-	-	+	-	-	-	-	-	1	+	+
Zinc and secondary molybdenum transporter																									
HIB_07090	*zit*B	-	-	+	-	-	-	-	-	-	-	-	-	-	+	+	-	-	-	-	+	+	5	+	+
HI1470	*mol*C	-	-	+	-	-	-	-	-	-	-	-	-	-	-	+	-	-	-	-	-	-	2	+	-^d^
HI1472	*mol*A	-	-	-	-	-	-	-	-	-	-	-	-	-	-	-	-	-	-	-	-	-	0	+	-^d^
*hmg* locus involved in LOS glycoform																									
HI0871	*sia*A	-	-	-	-	-	-	-	-	-	-	-	-	-	-	-	-	-	-	-	-	-	0	+	+
HI0872	*wba*P	-	-	-	-	-	-	-	-	-	-	-	-	-	-	-	-	-		-	-	-	0	+	+
No. of CDSs present		0	2	5	0	1	0	1	0	1	0	1	0	1	3	5	3	1	0	0	3	1		10	8

^a^Locus tags were based upon the genome of Rd Kw20 except HIB_07090 is based on Hib 10810. For PCR targeting the contiguous loci, only the first locus is shown. Full information of the target genes, amplicon size and PCR conditions is described in Additional file 1.

^b^Abbreviations: PTS, 6-pyruvoyl tetrahydrobiopterin synthase in folate biosynthesis.

^c^Total number of Hif clinical isolates (excluding KR494) with genes that were absent in Hif KR494.

^d^CDSs that were present in Rd Kw20 but not in NTHi 3655 or Hib MinnA.

^+^PCR positive (CDS present); ^-^PCR negative (CDS absent).

### Histidine biosynthesis defect and kanamycin resistance of Hif KR494

To confirm the relevance of the genetic findings, a series of functional experiments were performed. Since histidine is crucial for bacterial growth, we wanted to know whether the Hif gene deletions of the *his* operon would interfere with histidine biosynthesis and cell growth. We performed a histidine auxotrophic assay and found that all Hif isolates including KR494 were defective in growth when cultured in histidine-depleted medium (w/o-His) (Additional file 5). In contrast, the isolates grew well in histidine-supplemented (w-His) medium, whereas the positive controls Hib MinnA and NTHi 3655 readily grew in both w-His and w/o-His media. Our experiments thus showed that the inability to catalyze the *de novo* biosynthesis of histidine made Hif KR494 and other Hif isolates dependent on an external histidine source, excluding the possibility of any alternative histidine synthesis pathway.

Since *H. influenzae* kanamycin resistance is rarely reported, we also tested the Hif isolates for kanamycin susceptibility. We found that isolates containing the KNTase homologue (HifGL_000799-HifGL_000800) had kanamycin MIC of 4 μg/ml that was 4-fold higher than the Hib MinnA (1 μg/ml) and 8-fold higher than NTHi 3655 (0.5 μg/ml), which both lacked the kanamycin resistance genes (Additional file 5).

### Multiple genome alignments of closely-related human *Haemophilus*spp

**Figure 5:**
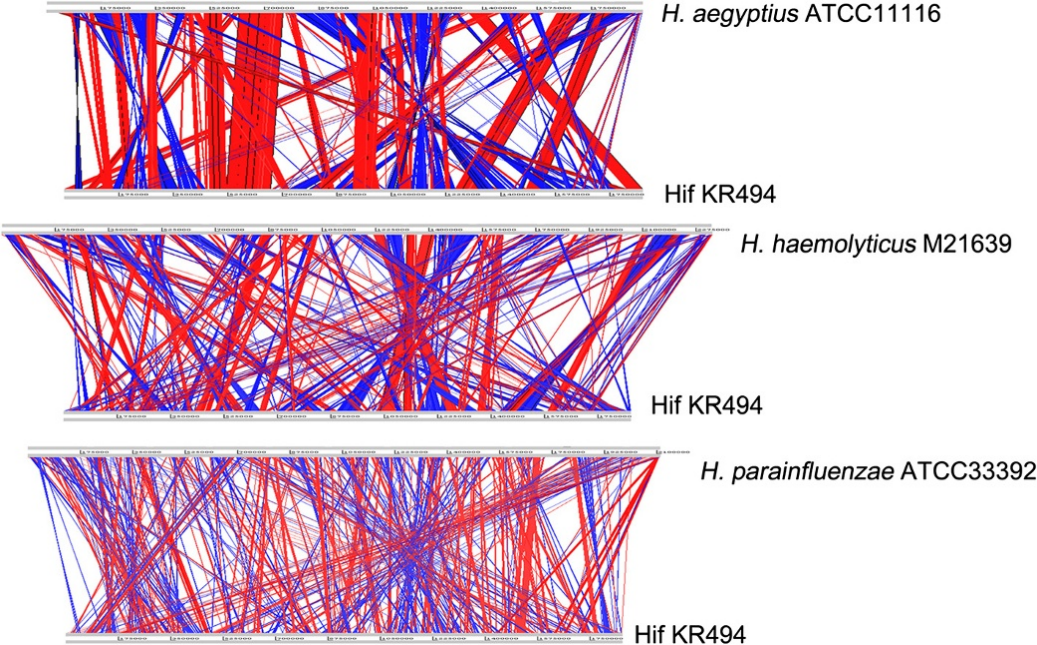
**A cross species genomic comparison of** ***H. influenzae*** **type f KR494 and human** ***Haemophilus*** **spp.** ACT view of synteny between genomes of Hif KR494 and *H. aegyptius* ATCC11116 (upper panel), *H. haemolyticus* M21639 (middle) and *H. parainfluenzae* ATCC33392 (lower panel). Respective genome designations are indicated on the right hand side of each genome line. Direct and inverted synteny between individual ORF (not indicated here) of the comparing genomes are shown in red and blue, respectively.

*Haemophilus influenzae*, *H. aegyptius* and *H. haemolyticus* belong to the cluster of ‘*Haemophilus sensu stricto’* (*Hss*) [62, 63], whereas *H. parainfluenzae* is located in a distinct cluster called *Parainfluenzae,* but is the closest neighbor species to the *Hss* group. Importantly, the *Hss* and *Parainfluenzae* groups share the same host, and thus are functionally closely related. Taking into account that a number of Hif KR494 unique genes are exclusively homologous to *H. aegyptius, H. haemolyticus* and *H. parainfluenzae*, a cross species genomic comparison was performed with these selected strains (Table 2). ACT alignment revealed an extensive divergence in gene order (Figure 5), indicating several gene rearrangements between the genomes of Hif KR494 and the related species. The Hif KR494 genome had a moderate level of synteny with the *H. aegyptius* ATCC 11116, but less with *H. haemolyticus* M21639 or *H. parainfluenzae* ATCC 33392. At a cut-off of 85% protein sequence similarity, a high number of genes from Hif KR494, that is, 1487 CDSs (85.4% of the total CDSs) were shared with *H. aegyptius*. This was reduced to 1451 CDSs (83.3%) with *H. haemolyticus* and 1366 CDSs (78.4%) with *H. parainfluenzae* (Table 2)*.* Our pangenomic data analyses thus implied a higher genome similarity between Hif and *H. aegyptius* than with *H. haemolyticus* and *H. parainfluenzae*, respectively. This picture confirmed previous phylogenetic studies on human *Haemophilus* spp. [62, 63]. In total, 1295 CDSs of Hif KR494 (74.3%) were in common with the related species, whereas 190 CDSs were less conserved mainly within the RgD_F_s (Additional file 6 and Additional file 7). Finally, a global pangenomic analysis revealed that 133 of the Hif KR494 unique genes were less conserved or absent in the *H. influenzae* reference strains, *H. aegyptius, H. haemolyticus* and *H. parainfluenzae* (Table 2), which represents the universal unique genome of Hif KR494 (Additional file 8).

## Discussion

To define the genomic factors that may contribute to the virulence traits of the emerging pathogen *H. influenzae* type f, we initially conducted a pangenomic analysis with three other complete genomes representing encapsulated and nontypeable *H. influenzae*. Since Hif isolates are suggested to be clonal [11, 18, 64], we used the recently sequenced Hif KR494, a necrotizing myositis isolate, as a primary model to identify genes potentially associated with virulence. We show that the divergence in the Hif KR494 genome is likely due to small-scale genetic rearrangements involving both gene acquisition (insertion) and gene loss in addition to some minor inversions. Importantly, the majority of genes identified as essential for the pathogenicity of *H. influenzae* were conserved and intact in Hif KR494 [65]. These include genes implicated in nutrient acquisition, LOS biosynthesis/modification and oxidative stress responses. Genes encoding for proteins mediating interactions with airway epithelial cells were also conserved [66, 67], reassuring Hif-dependent adhesion and subsequent colonization of the human host. Metabolic and growth requirements for *H. influenzae* have been very well studied, but solely based upon the strain Rd Kw20. Those analyses revealed at least 461 metabolic reactions operating on 367 internal metabolites and 84 external metabolites [68, 69]. Given that the Hif KR494 genome contains most but not all of the metabolic enzymes of Rd Kw20, we postulate similar metabolic machinery in Hif. A serotype-specific metabolism remains, however, to be determined.

Gene acquisition in *H. influenzae,* in particular the nontypeable strains, has been associated with bacterial genetic adaptation and is mainly attributed to horizontal DNA transfer [26, 34–36]. Like other mucosal pathogens, *H. influenzae* is naturally competent, and the ability to take up DNA is facilitated by the presence of DNA sequence uptake signals (USSs) [70]. There are 1496 USSs identified in the Hif KR494 genome (1566 in Hib 10810, 1471 in Rd Kw20, 1482 in NTHi 3655). The established mechanisms of horizontal DNA transfer in *H. influenzae* include transduction and infection by *Haemophilus* bacteriophages, DNA transformation, transconjugation of the ICE*Hin*1056 family and, finally, integration of genetic islands [54–56, 71–74]. We identified a Hif accessory genome that in several aspects, *i.e.*, surface structure, energy conversions and metabolic pathways, may contribute to the unique features of type f strains (Figure 3B). While the majority of known *H. influenzae-*associated virulence genes were conserved, the Hif genome contained additional putative virulence genes that were not identified in the *H. influenzae* reference strains. Several of these unique Hif KR494 CDSs had limited homology to the published *H. influenzae* accessory gene pool and exhibited atypical GC content (31.5%-48.5%) (Table 3). This clearly suggested acquisition through horizontal gene transfer. Notably, unique genes with slightly altered GC content may have been acquired from species with GC content similar to that of *H. influenzae.* The unique genes may have been introduced to the Hif genome through direct DNA uptake, transposed as a composite DNA, via prophage infection or integration as genetic islands. The presence of adjacent prophage islands and the abundance of mobile genetic elements and USS sites accompanying the unique genes suggest such events, mainly at RgD_F_s (Table 3). This implies that the Hif KR494 genome had a relatively uncomplicated gene acquisition mechanism that is not dependent on plasmids nor ICE elements since the latter two DNA components were not found in the Hif KR494.

Gene duplications have been shown to affect pathogenicity in some *H. influenzae* strains. Hib variants containing additional capsule loci are generally more virulent and the pathogenicity has been suggested to be proportionate to the gene copy number and amount of capsule deposited at the surface [75, 76]. Therefore, Hif KR494 genes that are associated with gene duplication were carefully determined. We postulate that a multiplication of metabolic genes may improve the Hif metabolism and energy production to enhance fitness during infection as described for several pathogenic fungi and the genetically related *H. influenzae* biogroup *aegyptius* and *H. aegyptius*[65, 77–79]. Additional paralogous genes associated with virulence such as the Sap transporter, fimbriae, heme transfer proteins and kanamycin resistance proteins may also increase virulence, and needs to be further studied.

We found a “duplication” of the Sap transporter, *i.e.*, SapABCDFZ and Sap2ABCDF, that may enhance the bacterial resistance against AMPs, increase the heme acquisition, and promote homeostasis in potassium uptake and interactions with epithelial cells [38, 39, 80]. The specific type of AMPs species targeted by the periplasmic solute binding protein Sap2A (a *H. parainfluenzae* SapA homologue) in Hif*,* is unknown and may be different from the *H. influenzae* SapA due to sequence heterogeneity.

The co-existence of different fimbriae types has been reported in *H. aegyptius* and *H. influenzae* biogroup *aegyptius* (conjunctivitis and BPF clones) [29, 41, 44]. NTHi and non-f capsulated *H. influenzae*, however, have only a single type of fimbriae locus (*hif*). Thus, this is the first report suggesting the presence of two distinct fimbriae loci (*hif* and *aef*) in Hif, resulting in a genotype similar to *H. aegyptius*. Although the role of Aef in virulence is not fully clear, the functionality of HifACDE fimbriae in Hif strains, which generally lack the chaperon subunit HifB may be compensated by the Aef3abcdef [29, 44]. The existence of Aef3 in the Hif genome offers an explanation to the haemagglutination phenotype of Hif that was observed despite the absence of the subunit *hif*B [12]. Interestingly, it is suggested that abundant pili/fimbriae (Hif and Aef) facilitate/promote initial colonization of the human nasopharynx, but are down-regulated prior to subsequent systemic invasion to prevent immune recognition [43].

Topology analysis with PSORTb suggested that the heme-binding protein HifGL_001444 is a periplasmic protein (data not shown). It may function as an alternative periplasmic transporter in addition to the previously described HbpA and HipA/DppA facilitating the transport of heme/iron across the periplasm to the cytoplasmic membrane transporter [49]. Moreover, the region spanning amino acid residues 52 and 322 comprises domain SBP_bac_8 (Pfam 13416), suggested that this protein belongs to the AfuA family (COG1840), a periplasmic component of ABC-type Fe^3+^ transport system. However, at the protein level HifGL_001444 shares only 33% of sequence homology with the AfuA of Rd Kw20. In addition, the HutZ (HifGL_0016364) homologue in *Vibro cholerae* was proposed as a heme storage protein and important for heme trafficking across the membrane to heme-containing proteins [81]. Thus, both HifGL_001444 and HutZ may confer a unique heme utilization machinery on Hif compared to other serotypes that lack these genes.

Intriguingly, all 20 Hif isolates screened in the present study contain the KNTase-related genes (HifGL_000799-HifGL_000800), and consequently are more resistant to kanamycin compared to Hib MinnA and NTHi 3655 that do not have these genes. This information is valuable for clinicians, since aminoglycosides are often used in the treatment of severe sepsis. More experiments are, however, needed to show the significance of KNTase-related genes in antimicrobial resistance.

Gene loss/deletion may also be beneficial for Hif although opposing the evolutionary force of gene acquisition. In fact, this phenomenon has been reported in *H. influenzae* biogroup aegyptius and other human pathogens such as *Francisella tularensis, Yersinia pestis* and *Shigella* spp., *Rickettsia* spp. and *Mycobacterium* spp. [77, 82–84]. Gene loss in microbial pathogens is principally caused by i) adaptation to a more specific niche of which certain gene products become unnecessary and, ii) inactivation/elimination of antivirulence genes (AVG) that is incompatible to newly acquired virulence factors [84]. However, the AVG concept has not yet been reported for *H. influenzae*. The majority of the deleted genes in Hif KR494 are not essential for establishing infection *in vivo*[65]. The Hif KR494 genome lacked three putative virulence genes involved in mouse pulmonary infection (*pdg*X, stress defense) and infant rat bactermia (*rfb*P and *rfb*B for LOS biosynthesis) [65].

The histidine biosynthesis pathway is of particular interest since it cannot be found in Hif KR494 or other Hif isolates examined in the present study (Tables 4 and 6). Since Hif is not a pathogen associated with acute otitis media this observation fits with the hypothesis that the histidine pathway is a survival strategy for NTHi isolates to cope with the limited histidine conditions in the middle ear [24]. The auxotrophic Hif phenotype, however, may not interfere with bacterial virulence as described in other *his* deficient species such as *Helicobacter pylori* 26695 and *Mycobacterium genitalium* G-37 [85, 86]. It is plausible that the histidine-rich environment in the throat may support initial colonization of Hif as reported in other *his*-negative auxotrophic throat commensals prior to its migration into systemic organs [24]. Since Hif KR494 was isolated from both blood and muscle tissue [9], its survival during subsequent invasive infection might depend on the uptake of exogenous histidine from surrounding niches. This is supported by the availability of free histidine or in the form of histamine and histidine-rich glycoproteins in blood and tissues [87–89]. We suggest that the absent histidine biosynthesis pathway may be one of the factors rendering Hif a less effective colonizer of the human airway, and may offer an explanation of why Hif is found in invasive disease rather than in respiratory tract infections. This speculation is consistent with the finding of gene loss events (*i.e.*, loss of genes involved in energy metabolism and nutrient transport) in BPF-related *H. influenzae* biogroup aegyptius HK1212. This particluar strain had a putative genome evolution as driving force towards a higher dependency on the host energy and metabolites for a secure adaptation to the host environment [77].

The quantities of molybdate are reported in the ~100-1000 nM range in whole blood [90]. For Hif, the high affinity ModABCE system (K_d_ = 10 nM-1 μM) might be preferable, whereas the low affinity MolABCD system (K_d_ = ~100 μM) becomes superfluous [60, 61]. Genes involved in the LOS biosynthesis were altered since the entire *hmg* responsible for different LOS glycoforms was deleted. Moreover, *sia*A encoded within the *hmg* locus is required for biofilm formation of NTHi otitis media isolates [59, 91]. Mucosal pathogens are generally protected by a biofilm that promotes local colonization, and consequently prevents detachment and transmission from the infection site [92]. While we cannot rule out the impact of *hmg* gene deletion for Hif virulence, we hypothesized whether the gene loss may cause defects in biofilm formation, aiding to bacterial systemic dissemination from mucosal sites, as seen in the hypervirulent *Neisseria meningitidis*[93].

In addition to Hif strains that are monophyletic regarding the 7 housekeeping genes used in MLST [18], both Rodriguez *et al*. and Watson *et al.*[11, 12] found a homogenous distribution of the known virulence genes (*hsf, hif, hap* and *lic*2BC). Based on these studies, it was suspected that Hif isolates are generally clonal. The analysis of the unique genomic features identified in the Hif KR494 genome in 20 clinical Hif strains confirmed this assumption (Tables 5 and 6). In addition, MLST showed that all Hif isolates tested from different parts of Sweden were of sequence type (ST) 124 [3]. Most clinical Hif strains displayed a near-perfect match to Hif KR494, varying only in one to four genes. Our result is also congruent with previous studies supporting the limited genetic diversity of serotype f despite being implicated in a wide variety of clinical severities and infection sites.

The Hif KR494 genome exerted a *H. aegyptius-*like genotypic characteristic, *i.e.*, 85.4% of the total Hif KR494 CDSs were homologous to *H. aegyptius* ATCC1116. This is proximate to the degree of similarity (85.8%) observed between Hif KR494 and Hib 10810, and was more conserved than Hid Rd Kw20 (83.7%). Although two prior phylogenetic investigations revealed that *H. aegyptius* ATCC1116 is genetically the closest *Haemophilus* species to *H. influenzae* based upon the nontypeable HK389 and typeable P1557 (serotype a) and P1227 (serotype b), Hif was not included in those studies and the precise relationship thus needs to be further elucidated [62, 63]. Nonetheless within the species of *H. influenzae*, phylogenetical analysis (based on MLST) by Meats *et al.*[18] together with a recent phylogenomic study (pairwise alignment of partial-genome sequences from 70 nontypeable and capsulated (a-f; except d) strains) revealed that serotype f is genetically closely related to serotype a and e [18, 74]. This is interesting since both serotype a and e were recently reported to be potentially invasive [4, 94, 95]. However, attempts to include type a and e in our current study was hampered by the absence of the reported genome sequences in public databases [74]. The genetical diversity of the Hif KR494 genome (Table 3), however, is limited to orthologs within the *Hin* subclade (*H. influenzae, H. aegyptius, H. parainfluenzae, H. haemolyticus, A. actinomycetemcomitans, P. multocida, M. succiniciproducens,* and *H. somnus*) of the *Pasteurellaceae* family. This may be explained by the findings of Redfield *et al*. [96], which revealed a DNA uptake specificity of the *Hin* subclade that is preferentially dependent on *H. influenzae* USS consensus sequence.

## Conclusions

The comparative analyses have identified unique features of the Hif KR494 genome that may increase the understanding of Hif pathogenesis. Gene rearrangements involving inversion, insertion and deletion were evident despite a large similarity in genomic organization between Hif and other previously sequenced serotypes. Our analysis resulted in a wide compilation of gene functions unique to Hif. The gene products involved in metabolism and virulence that are not found in other serotypes may also contribute to the Hif pathogenicity associated with invasive disease. The *in silico* analysis, however, did not make it possible to determine the specific virulence factors that may explain differences between the analyzed *Haemophilus* species. It remains to elucidate whether these newly discovered Hif genes can be used as biomarkers for serotype differentiation or targets for antimicrobial drug design.

## Authors’ information

YCS, FR and KR are research scientists at Medical Microbiology, Department of Laboratory Medicine Malmö, Lund University, Jan Waldenströms gata 59, SE-205 02 Malmö, Sweden. FH is a bioinformatics student at Institute of Computer Science, Department of Mathematics and Computer Science, Friedrich-Schiller-University of Jena, PF 07737, Jena, Germany.

## Electronic supplementary material

Additional file 1: **List of primers and PCR conditions used in present study.** (PDF 13 KB)

Additional file 2: **ACT view of multiple genome alignment among human-related** ***Haemophilus*** **spp.** Respective genome designations are indicated on the right hand side of each genome line. Forward (+) and complement (−) strands of individual genomes are indicated in the grey genome lines. Genomes are shown in full length and drawn to scale. Direct and inverted synteny between individual ORF (not indicated here) of the compared genomes are shown in red and blue, respectively. The level of amino acid similarity is represented by color shading with ascending saturation and indicates higher similarity. (PDF 243 KB)

Additional file 3: **List of loci encoded at RgD**_**F**_ **s of the Hif KR494 genome.** (PDF 7 KB)

Additional file 4: **Predicted genetic islands of serotype f (Gif**_**KR494**_ **) defined in the Hif KR494 genome.** (PDF 10 KB)

Additional file 5: **Histidine auxotrophic and antibiotics susceptibility assay.** (PDF 54 KB)

Additional file 6: **Cross species genomic comparative of the closely-related human** ***Haemophilus*** **spp.** A cross species genomic comparison of *H. influenzae* type f KR494 and human *Haemophilus* spp (*H. aegyptius* ATCC 11116, *H. haemolyticus* M21639 and *H. parainfluenzae* ATCC 33392). COG distribution and functionality classification of Hif KR494 CDSs that is (A) commonly shared and (B) unique CDSs in regards to the related *Haemophilus* spp. (PDF 107 KB)

Additional file 7: **Map of RgD**_**F**_ **in the** ***H. influenzae*** **type f KR494 genome in relative to the closely-related human** ***Haemophilus*** **species.** Circular representation of protein conservation between Hif KR494 and the reference species was visualized using DNA plotter. From the outside in, the outer circle shows the genome length of Hif KR494 with position markers. The second circle shows the total ORFs of KR494 genome predicted on both forward and reverse strands. Common and unique ORFs in relative to the reference species are colored in blue and magenta, respectively. Phage-related ORFs are marked in yellow and orange. The third to fifth circles represent the distribution of individual ORF with high homology (≥85% similarity) (in red) to the corresponding ORF in reference species *H. aegyptius* ATCC11116, *H. haemolyticus* M21639 and *H. parainfluenzae* ATCC33392, respectively. Gaps between the conserved ORFs represent RgD_F_ between Hif KR494 and the compared species, and were denoted as RgD_F_1 to RgD_F_7 (marked with green lines). GC plot and GC skew of the Hif KR494 genome are shown in the sixth and seventh circle, respectively. The genome of Hif KR494 was less conserved with *H. aegyptius, H. haemolyticus* and *H. parainfluenzae* at the RgD_F_1, 2, 3, 5, 6 and 7. The RgD_F_7 comprises *xyl*FGH and *xyl*AB operons (HifGL_000770-HifGL_000777) that are involved in xylose uptake and metabolism through the pentose phosphate pathway. This indicated that *H. aegyptius, H. haemolyticus* and *H. parainfluenzae* lacked a xylose metabolism system that is, however, conserved in *H. influenzae*. (PDF 323 KB)

Additional file 8: **Total genomic comparison of Hif KR494 with** ***H. influenzae*** **reference strains,** ***H. aegyptius, H. haemolyticus*** **and** ***H. parainfluenzae.*** Unique genes (133 CDSs) of Hif KR494 that consistently lacked homology with any of the aligned species were delineated based on the COG database. Notably, when genes of unknown function were excluded, most of the universal unique CDSs of Hif KR494 were phage-related products, followed by extracellular structures. The data represent the universal gene feature of Hif KR494. (PDF 77 KB)

## Abbreviations

ACT: Artemis comparative tools
AVG: Antivirulence gene
CDS: Coding sequence
COPD: Chronic obstructive pulmonary diseases
COG: Cluster of orthologous groups
Hib: *H. influenzae* type b
Hid: *H. influenzae* type d
Hif: *H. influenzae* type f
LOS: Lipooligosaccharide
MIC: Minimal inhibitory concentration
NTHi: Nontypeable *H. influenzae*
ORF: Open reading frame
RgDF: Hif-region of difference
TAA: Trimeric autotransporter.
