# Supplementary material for: Comparative genomic analysis reveals distinct genotypic features of the emerging pathogen Haemophilus influenzae type f
Source: BMC Genomics. 2014 Jan 18;15(1):38. doi: 10.1186/1471-2164-15-38 (PMC3928620; doi:10.1186/1471-2164-15-38)
Supplement: Supplementary file 1 — Additional file 1: List of primers and PCR conditions used in present study. (PDF 13 KB) [file 12864_2013_7004_MOESM1_ESM.pdf]

**Additional file 1. List of primers and PCR conditions used in present study.**

| Locus tag[a] | Function/description | Primer pair [b] | Forward primer [b] | Reverse primer [b] | Amplicon size (bp) | PCR condition[c] |
|---|---|---|---|---|---|---|
| HifGL_000176 | Cell-wall associated hydrolase on genetic island | Hif_U1 | CCGCACTTAGCTACCCGGCAATGCGTCT | GTCTGAGTAGGCAAGACAGGAAAGTGAG | 2919 | 52°C/ 4 min |
| HifGL_000310 | Glutathione S-transferase (GST) domain-containing protein | Hif_U2 | ATGAAACTTTGGTACTCCACTACCAGCCCG | TCTGCCGCCCAAGCGGTTAAATTTGGAA | 572 | 54°C/ 1 min |
| HifGL_000674-HifGL_000685 | RgD$_F$ 1: *sap*2ABCDF | Hif_U3 | GTGATTATCTTATTATTGATTCACACTTTG | TGATTCTGTATTGAGCGTAAGTGGTGAC | 2852 | 54°C/ 4 min |
| HifGL_000799-HifGL_000800 | Homologues of *Staphylococcus aureus* kanamycin nucleotidyltranserase (KNTase) | Hif_U4 | ATATTTTCAACCAATTGAGCGTGAGTCA | ATGCGATCAAATAATCGTTGGCTACAACGT | 670 | 54°C/ 1 min |
| HifGL_000834-HifGL_000849 | RgD$_F$2: Duplication of gene cluster involved in sugar and amino acid transport and metabolism | Hif_U5 | GATTGAACAAAATAGAGCAAATAACTGG | TTGCTCATTTCTTAATGACTAACGGA | 2526 | 50°C/ 3 min |
| HifGL_000837 | Trimeric autotransporter (TAA) | Hif_U6 | ATGCATTCATCTTCACATGCACTAACAG | TTCGCTTTCCATTTCAGCTTGTTTATGCAGTT | 1041 | 54°C/ 1 min |
| HifGL_000989-HifGL_000995 | RgD$_F$3: *aef*3ABCDEF | Hif_U7 | TACATCTGGACAAGTGGTCGAAGGGTTT | GCGACCTTCTAATTCTCCACCTTCAA | 4299 | 52°C/ 5 min |
| HifGL_001007-HifGL_001026 | RgD$_F$3: Small prophage island | Hif_U8 | TTTATCTCCCACTCTTTATACTCTTCTT | TGCTGTATTGGCTAAATATTCACTGTGA | 3840 | 54°C/ 4 min |
| HifGL_001350-HifGL_001353 | RgD$_F$4: Duplication of *rnf* electron transport complex | Hif_U9 | AATCGGAAGAATACGCCCTCAAGAT | TTCGCCCGCAGTCATTACATCTACAT | 2669 | 54°C/ 3 min |

| | | | | | | |
|---|---|---|---|---|---|---|
| HifGL_001363-HifGL_001379 | $RgD_F4$: Prophage island | Hif_U10 | TTTACTCGGGGTGGTTCATCCCATACTG | AAATTGCCGTGGTAGGATTGAGCTGTAG | 4567 | 52°C/ 5 min |
| HifGL_001431 | Trimeric autotransporter (TAA) | Hif_U11 | ATGGCATTGGTAAGTAGTGCGGTTT | CTAAAACCCAAAACTTACCCCTGTTGCA | 1896 | 54°C/ 2 min |
| HifGL_001444 | $Fe^{3+}$ ABC superfamily; ATP-binding cassette transporter | Hif_U12 | GTGCTTGGTTGCGGCTTATTAGGATT | CTAAATCCAAATCGGCTTTCGTGATA | 2609 | 52°C/ 3 min |
| HifGL_001463 | Hypothetical protein of unknown function | Hif_U13 | ATGGCAAATGTAATAAAGATTCCAGCAAAA | TTAATAATTGCCTTTTTTAATTAATGTATC | 450 | 54°C/ 40 sec |
| HifGL_001635 | $RgD_F6$: Type II restriction enzyme *Hinf*I | Hif_U15 | ATGAATGATGAAATTTCCGACCTAA | TATTATCGGGACTTTCTGAAAGGTT | 786 | 54°C/ 1 min |
| HifGL_001636 | $RgD_F6$: Type f-specific adenine-specific methyltransferase *Hinf*I | Hif_U14 | AATTCCGAATGAAAGTATTGATTTAATC | TTATTGGTTAGTGTATTCATATCTTAAACT | 1009 | 54°C/ 1 min |
| HifGL_001664 | Heme-binding protein HutZ | Hif_U16 | ATGAACTTAAATCAGGAGAAAAGTGTACGT | TTAATTCTTGGTGTCCTTCAACCAAGTGAAC | 276 | 54°C/ 30 sec |
| HifGL_001672 | Lipoprotein of unknown function | Hif_U17 | ATGAAGGAATTTACAATGAAATTTAATAAATC | TTATTTTTGCTCTGAAACTAAACCAATTTG | 609 | 54°C/ 1 min |
| HI0468 | *his*G of histidine biosynthesis operon | Hif_M1 | ATGACAAACACAACAATGCAACCAAACC | TTACTCCATCATCTTCTCAATTGGTAACA | 912 | 54°C 1 min |
| HI0469 | *his*D of histidine biosynthesis operon | Hif_M2 | CAAAGTCATTATGCGTCCAGTCCAACA | ATTGCTCAGCTTCTGCCATCACTTCCA | 1187 | 51°C/ 70 sec |
| HI0470 | *his*C of histidine biosynthesis operon | Hif_M3 | ATGACAATCACAACTTTATCCCGACAA | TTTGTAAATGCAATGTCTTATTCTGGTC | 985 | 54°C/ 1 min |
| HI0472 | *his*H of histidine biosynthesis operon | Hif_M4 | ATGATAAACATCACGATTATAGACACAGG | TTTTAGCAACAACGCACCATTTTTACC | 573 | 51°C/ 1 min |

| | | | | | | |
|---|---|---|---|---|---|---|
| HI1024 | *ula*D involved in anaerobic fermentation of L-ascorbate | Hif_M5 | ATGGGAAAACCTTTATTACAAATCGCT | TATATCCAAAATTGCCCTATTTTCTGT | 677 | 54°C/ 1 min |
| HI1190 | 6-pyruvoyl tetrahydrobiopterin synthase in folate biosynthesis | Hif_M6 | TTTAAAATTTCCAAAGAATTTAGCTTT | TTACTCCTGATACTCGCAAAATGAAGT | 423 | 54°C/ 40 sec |
| HIB_07090 | Zinc transporter ZitB | Hif_M7 | ATGAAAGAAGTTTCTCTAGTAAAAAATTA | TTAATGAGAATGATCATTCGGCAATGA | 678 | 54°C/ 1 min |
| HI1470 | *mol*C of secondary molybdenum transporter | Hif_M8 | ATAAAGCGCTCTCCGTGGAAAATCTAG | TTACTTTAATAACGTTTTATACAAGGGAAC | 758 | 54°C/ 1 min |
| HI1472 | *mol*A of secondary molybdenum transporter | Hif_M9 | ATGAAACTTAAATCATTGTTAATTGCCT | CTATCTGGCTGCATTGTCGGGTTTGTAA | 1056 | 54°C/ 1 min |
| HI0871 | *sia*A of *hmg* locus involved in LOS biosynthesis | Hif_M10 | ATAATTATCAGTAATATGAGGATGTTATT | CTTTTGGGTATACATATACAGCCGTTGAG | 826 | 54°C/ 1 min |
| HI0872 | *wba*P of *hmg* locus involved in LOS biosynthesis | Hif_M11 | TTACCTGTTTACGAAGTGGATGATAG | ATAGCAATATCATTCCAAAGTGACCA | 1247 | 54°C/ 1 min |

[a] Locus tagged with prefix "HifGL" represents CDS from Hif KR494, "HI" is from Hid RD Kw20 and "HIB" is from Hib 10810. Primers were designed based on the indicated loci.

[b] Primers denoted with prefix "Hif_U" were used to screen Hif unique genes, whereas those denoted with "Hif_M" were used for identifying missing genes.

[c] The annealing temperature/ elongation times are indicated. All PCRs were performed with 35 cycles, standard concentrations of reagents and buffer, and with normal *Taq* DNA polymerase.
