# Supplementary material for: Comparative genomic analysis reveals distinct genotypic features of the emerging pathogen Haemophilus influenzae type f
Source: BMC Genomics. 2014 Jan 18;15(1):38. doi: 10.1186/1471-2164-15-38 (PMC3928620; doi:10.1186/1471-2164-15-38)
Supplement: Supplementary file 2 — Additional file 2: ACT view of multiple genome alignment among human-related Haemophilus spp. Respective genome designations are indicated on the right hand side of each genome line. Forward (+) and complement (−) strands of individual genomes are indicated in the grey genome lines. Genomes are shown in full length and drawn to scale. Direct and inverted synteny between individual ORF (not indicated here) of the compared genomes are shown in red and blue, respectively. The level of amino acid similarity is represented by color shading with ascending saturation and indicates higher similarity. (PDF 243 KB) [file 12864_2013_7004_MOESM2_ESM.pdf]

**Additional file 2:**

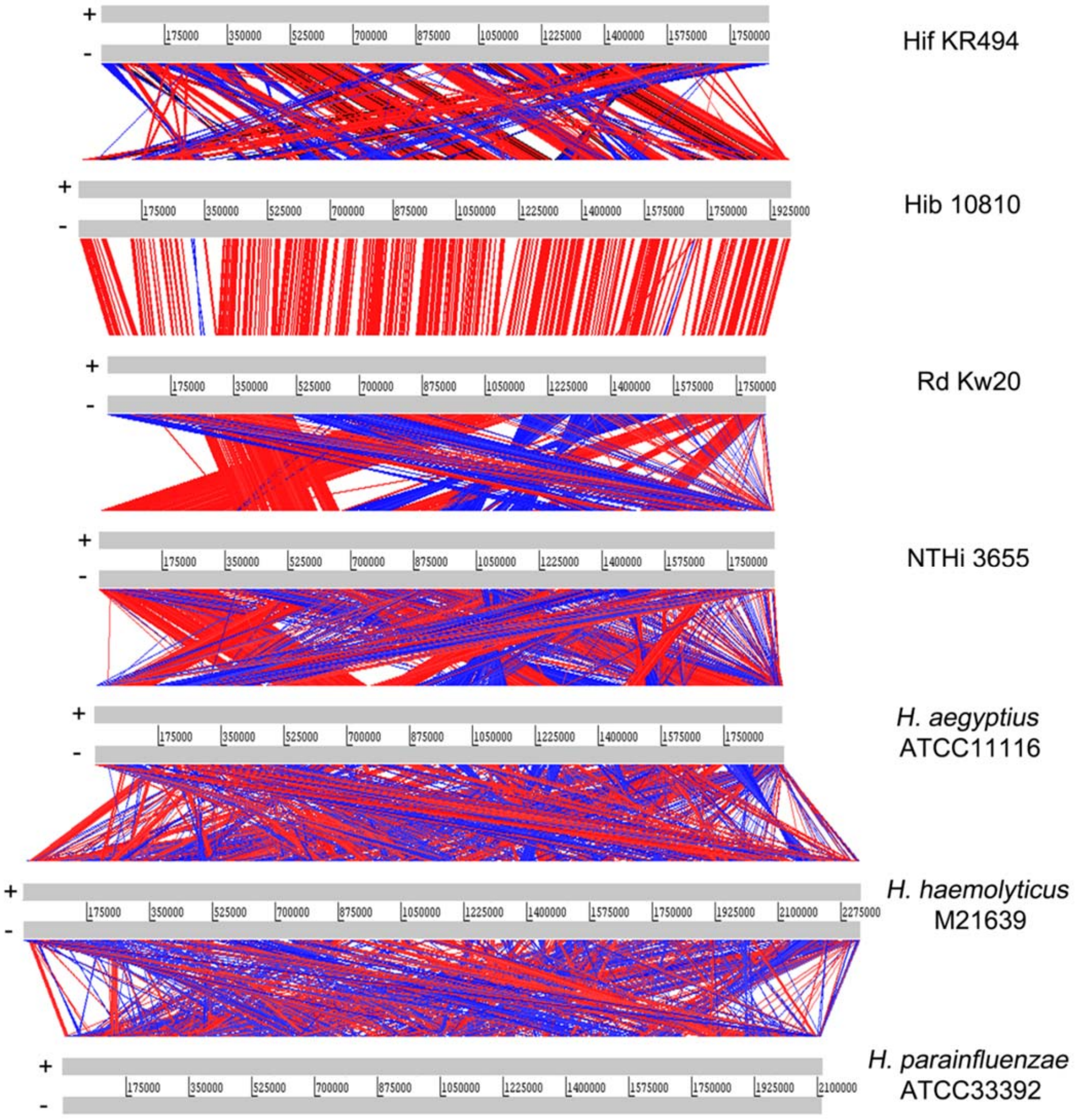


**ACT view of multiple genome alignment among human-related *Haemophilus* spp.** Respective genome designations are indicated on the right hand side of each genome line. Forward (+) and complement (-) strands of individual genomes are indicated in the grey genome lines. Genomes are shown in full length and drawn to scale. Direct and inverted synteny between individual ORF (not indicated here) of the comparing genomes are shown in red and blue, respectively. The level of amino acid similarity is represented by color shading with ascending saturation and indicates higher similarity.
