# Supplementary material for: Comparative genomic analysis reveals distinct genotypic features of the emerging pathogen Haemophilus influenzae type f
Source: BMC Genomics. 2014 Jan 18;15(1):38. doi: 10.1186/1471-2164-15-38 (PMC3928620; doi:10.1186/1471-2164-15-38)
Supplement: Supplementary file 3 — Additional file 3: List of loci encoded at RgD F s of the Hif KR494 genome. (PDF 7 KB) [file 12864_2013_7004_MOESM3_ESM.pdf]

Additional file 3: List of loci encoded at RgD_Fs of the Hif KR494 genome.

RgD_F	Locus tag	Average G+C content (%)
1	HifGL_000650-HifGL_000714	38.86
2	HifGL_000834-HifGL_000849	35.75
3	HifGL_000989-HifGL_001032	38.22
4	HifGL_001349-HifGL_001379	39.28
5	HifGL_001545-HifGL_001588	34.75
6	HifGL_001625-HifGL_001636	38.19
7	HifGL_000770-HifGL_000777	35.03
