# Supplementary material for: Comparative genomic analysis reveals distinct genotypic features of the emerging pathogen Haemophilus influenzae type f
Source: BMC Genomics. 2014 Jan 18;15(1):38. doi: 10.1186/1471-2164-15-38 (PMC3928620; doi:10.1186/1471-2164-15-38)
Supplement: Supplementary file 4 — Additional file 4: Predicted genetic islands of serotype f (Gif KR494 ) defined in the Hif KR494 genome. (PDF 10 KB) [file 12864_2013_7004_MOESM4_ESM.pdf]

Additional file 4: Predicted genetic islands of serotype f (Gif_{KR494}) defined in the Hif KR494 genome.

Gif_{KR494}[a]	Nucleotide position[b]	Average G+C content (%)	RgD_F	Locus encoded on Gif_{KR494}
1	131904-137514	38.46		HifGL_000131-HifGL_000133
2[c]	179248-182347	48.12		HifGL_000176
3	182804-184536	49.61		-
4	188683-195050	35.36		HifGL_000181-HifGL_000186
5	221783-223516	35.74		HifGL_000216-HifGL_000219
6	223955-225116	44.11		HifGL_000220
7	225419-227393	32.89		HifGL_000221-HifGL_000222
8[c]	235628-238425	49.64		HifGL_000233
9	239095-240826	49.83		-
10	413890-419275	39.36		HifGL_000400-HifGL_000402
11[P]	694054-719928	42.77	1	HifGL_000683-HifGL_000714
12	1161265-1163347	39.62		HifGL_001145-HifGL_001146
13[P]	1380225-1389278	39.57	4	HifGL_001363-HifGL_001379
14[c]	1437198-1440111	49.25		HifGL_001417
15	1440538-1442123	50.74		-
16	1615261-1621036	38.07	5	HifGL_001582-HifGL_001586
17[c]	1629069-1632003	49.11		HifGL_001593
18	1632663-1634411	49.69		-
19[c]	1662327-1665306	48.58		HifGL_001616
20	1665744-1667511	49.50		-
21	1671794-1677252	37.28	6	HifGL_001620-HifGL_001626
22[c]	1718986-1721872	49.11		HifGL_001665
23	1722542-1724121	51.60		-

[a] Genetic island prediction was performed based on sequence composition by using software IslandPath-DIOMB [1] and SIGI-HMM [2]. Prediction was also done based on multi-genome comparison with Island Picker and IslandViewer [3].

[b] Nucleotide position was based on the forward strand.

[c] Gif_{KR494} with similar sequence and contain the identical CDS (HifGL_00176, HifGL_000233, HifGL_001417, HifGL_001593, HifGL_001616 and HifGL_001665: cell-wall associated hydrolase).

[P] Prophage island.

References

1. Hsiao W, Wan I, Jones SJ, Brinkman FSL: **IslandPath: aiding detection of genomic islands in prokaryotes.** *Bioinformatics* 2003, **19:**418-420.
2. Waack S, Keller O, Asper R, Brodag T, Damm C, Fricke WF, Surovcik K, Meinicke P, Merkl R: **Score-based prediction of genomic islands in prokaryotic genomes using hidden Markov models.** *BMC Bioinformatics* 2006.**7:**142.
3. Langile MCI, Brinkman FSL: **IslandViewer: an integrated interface for computational identification and visualization of genomic islands.** *Bioinformatics* 2009, **25:**664-665.
