# Supplementary material for: Comparative genomic analysis reveals distinct genotypic features of the emerging pathogen Haemophilus influenzae type f
Source: BMC Genomics. 2014 Jan 18;15(1):38. doi: 10.1186/1471-2164-15-38 (PMC3928620; doi:10.1186/1471-2164-15-38)
Supplement: Supplementary file 5 — Additional file 5: Histidine auxotrophic and antibiotics susceptibility assay. (PDF 54 KB) [file 12864_2013_7004_MOESM5_ESM.pdf]

Additional file 5: Histidine auxotrophic and antibiotics susceptibility assay.

	Histidine auxotrophic				Kanamycin resistance	
	Gene[a] (*his*GCDH)	Growth[b] w/o-His[c]	Growth[b] w-His[c]	Growth[b] sBHIB[c]	Gene[a] (HifGL_000799)	MIC (µg/ml)
**Laboratory strain**						
NTHi 3655	+	+	+	+	-	<2.0
Hib MinnA	+	+	+	+	-	<0.5
**Clinical isolates**						
Hif						
KR494	-	-	+	+	+	>4.0
G19	-	-	+	+	+	>4.0
G20	-	-	+	+	+	>4.0
K238	-	-	+	+	+	>4.0
L11	-	-	+	+	+	>4.0
L16	-	-	+	+	+	>4.0
L21	-	-	+	+	+	>4.0
L22	-	-	+	+	+	>4.0
L24	-	-	+	+	+	>4.0
L25	-	-	+	+	+	>4.0
L29	-	-	+	+	+	>4.0
L45	-	-	+	+	+	>4.0
L50	-	-	+	+	+	>4.0
L59	-	-	+	+	+	>4.0
M1	-	-	+	+	+	>4.0
M10	-	-	+	+	+	>4.0
M14	-	-	+	+	+	>4.0
M29	-	-	+	+	+	>4.0
M54	-	-	+	+	+	>4.0
S208	-	-	+	+	+	>4.0
S229	-	-	+	+	+	>4.0

[a] Genes/operon were considered as present (+) or absent (-) according to results with PCR screening (main text Tables 5 and 6).

[b] Growth was considered as positive (+) when the OD_{600} value after 12 hr culture was >0.1, and negative (-) for $OD_{600} \leq 0.1$.

[c] w/o-His, histidine-depleted Herriot defined media; w-His, histidine (w/v=0.001%)-supplemented defined media; sBHIB, NAD and hemin (each at 10 µg/ml) supplemented BHI broth.
