# Supplementary material for: Comparative genomic analysis reveals distinct genotypic features of the emerging pathogen Haemophilus influenzae type f
Source: BMC Genomics. 2014 Jan 18;15(1):38. doi: 10.1186/1471-2164-15-38 (PMC3928620; doi:10.1186/1471-2164-15-38)
Supplement: Supplementary file 6 — Additional file 6: Cross species genomic comparative of the closely-related human Haemophilus spp. A cross species genomic comparison of H. influenzae type f KR494 and human Haemophilus spp (H. aegyptius ATCC 11116, H. haemolyticus M21639 and H. parainfluenzae ATCC 33392). COG distribution and functionality classification of Hif KR494 CDSs that is (A) commonly shared and (B) unique CDSs in regards to the related Haemophilus spp. (PDF 107 KB) [file 12864_2013_7004_MOESM6_ESM.pdf]

Additional file 6:

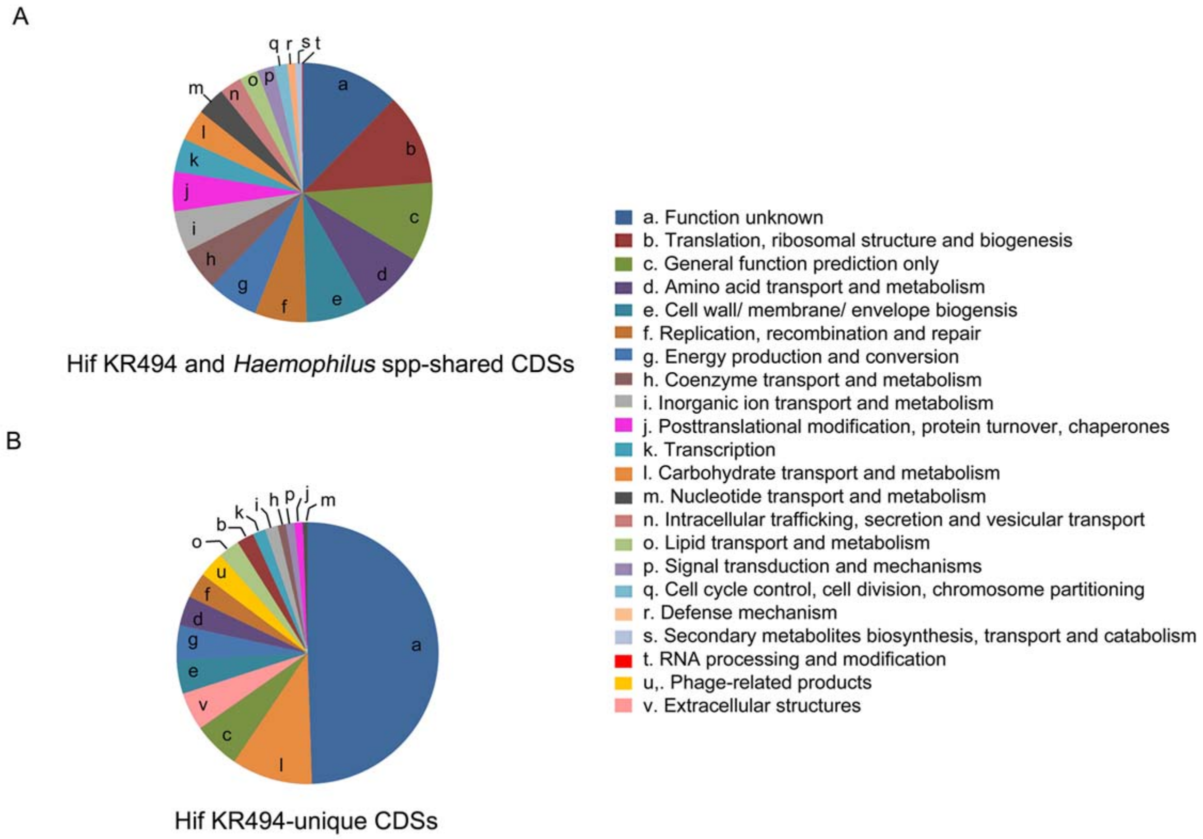

Additional file 6: Cross species genomic comparative of the closely related human *Haemophilus* spp. A cross species genomic comparison of *H. influenzae* type f KR494 and human *Haemophilus* spp (*H. aegyptius* ATCC 11116, *H. haemolyticus* M21639 and *H. parainfluenzae* ATCC 33392). COG distribution and functionality classification of Hif KR494 CDSs that is (**A**) commonly shared and (**B**) unique CDSs in regards to the related *Haemophilus* spp.
