# Supplementary material for: Comparative genomic analysis reveals distinct genotypic features of the emerging pathogen Haemophilus influenzae type f
Source: BMC Genomics. 2014 Jan 18;15(1):38. doi: 10.1186/1471-2164-15-38 (PMC3928620; doi:10.1186/1471-2164-15-38)
Supplement: Supplementary file 7 — Additional file 7: Map of RgD F in the H. influenzae type f KR494 genome in relative to the closely-related human Haemophilus species. Circular representation of protein conservation between Hif KR494 and the reference species was visualized using DNA plotter. From the outside in, the outer circle shows the genome length of Hif KR494 with position markers. The second circle shows the total ORFs of KR494 genome predicted on both forward and reverse strands. Common and unique ORFs in relative to the reference species are colored in blue and magenta, respectively. Phage-related ORFs are marked in yellow and orange. The third to fifth circles represent the distribution of individual ORF with high homology (≥85% similarity) (in red) to the corresponding ORF in reference species H. aegyptius ATCC11116, H. haemolyticus M21639 and H. parainfluenzae ATCC33392, respectively. Gaps between the conserved ORFs represent RgDF between Hif KR494 and the compared species, and were denoted as RgDF1 to RgDF7 (marked with green lines). GC plot and GC skew of the Hif KR494 genome are shown in the sixth and seventh circle, respectively. The genome of Hif KR494 was less conserved with H. aegyptius, H. haemolyticus and H. parainfluenzae at the RgDF1, 2, 3, 5, 6 and 7. The RgDF7 comprises xylFGH and xylAB operons (HifGL_000770-HifGL_000777) that are involved in xylose uptake and metabolism through the pentose phosphate pathway. This indicated that H. aegyptius, H. haemolyticus and H. parainfluenzae lacked a xylose metabolism system that is, however, conserved in H. influenzae. (PDF 323 KB) [file 12864_2013_7004_MOESM7_ESM.pdf]

Additional file 7

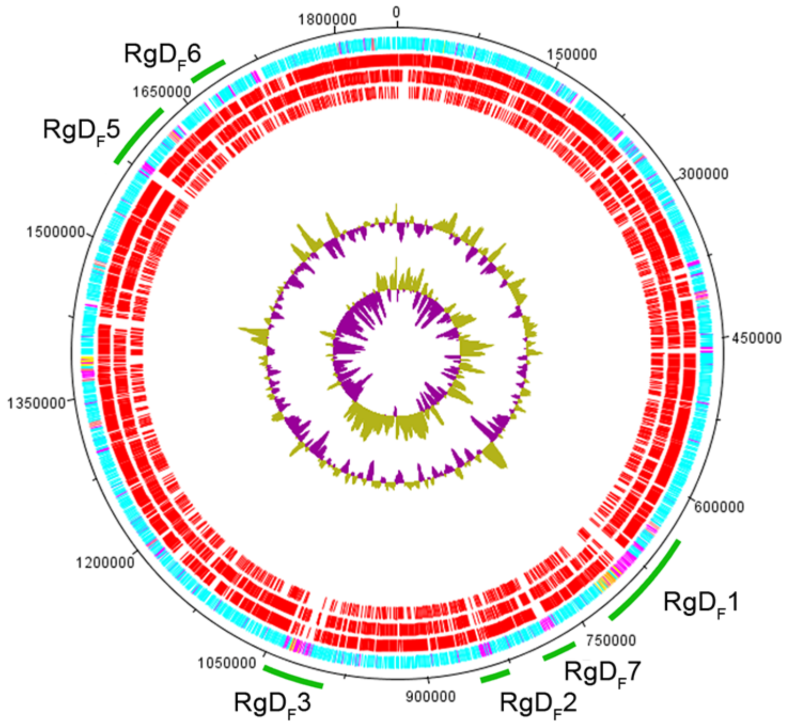

Map of RgD$_F$ in *H. influenzae* type f KR494 genome in relative to closely-related human *Haemophilus* species. Circular representation of protein conservation between Hif KR494 and reference species was visualized using DNA plotter. From the outside in, the outer circle shows the genome length of Hif KR494 with position markers. The second circle shows the total ORFs of KR494 genome predicted on both forward and reverse strands. Common and unique ORFs in relative to the reference species are colored in blue and magenta, respectively. Phage-related ORFs are marked in yellow and orange. The third to fifth circles represent the distribution of individual ORF with high homology (≥85% identity) (in red) to the corresponding ORF in reference species *H. aegyptius* ATCC11116, *H. haemolyticus* M21639 and *H. parainfluenzae* ATCC33392, respectively. Gaps between the conserved ORFs represent RD between Hif KR494 and the compared species and were denoted as RgD$_F$1 to RgD$_F$7 (marked with green lines). GC plot and GC skew of KR494

genome are shown in the sixth and seventh circle, respectively. Genome of Hif KR494 was less conserved with *H. aegyptius, H. haemolyticus* and *H. parainfluenzae* at the RgD_F1, 2, 3, 5, 6 and 7. RgD_F7 comprises *xyl*FGH and *xyl*AB operons (HifGL_000770-HifGL_000777) that are involved in xylose uptake and metabolism through the pentose phosphate pathway. This indicated that *H. aegyptius, H. haemolyticus* and *H. parainfluenzae* lacked a xylose metabolism system that is, however, conserved in *H. influenzae*.
