# Supplementary material for: Comparative genomic analysis reveals distinct genotypic features of the emerging pathogen Haemophilus influenzae type f
Source: BMC Genomics. 2014 Jan 18;15(1):38. doi: 10.1186/1471-2164-15-38 (PMC3928620; doi:10.1186/1471-2164-15-38)
Supplement: Supplementary file 8 — Additional file 8: Total genomic comparison of Hif KR494 with H. influenzae reference strains, H. aegyptius, H. haemolyticus and H. parainfluenzae. Unique genes (133 CDSs) of Hif KR494 that consistently lacked homology with any of the aligned species were delineated based on the COG database. Notably, when genes of unknown function were excluded, most of the universal unique CDSs of Hif KR494 were phage-related products, followed by extracellular structures. The data represent the universal gene feature of Hif KR494. (PDF 77 KB) [file 12864_2013_7004_MOESM8_ESM.pdf]

Additional file 8

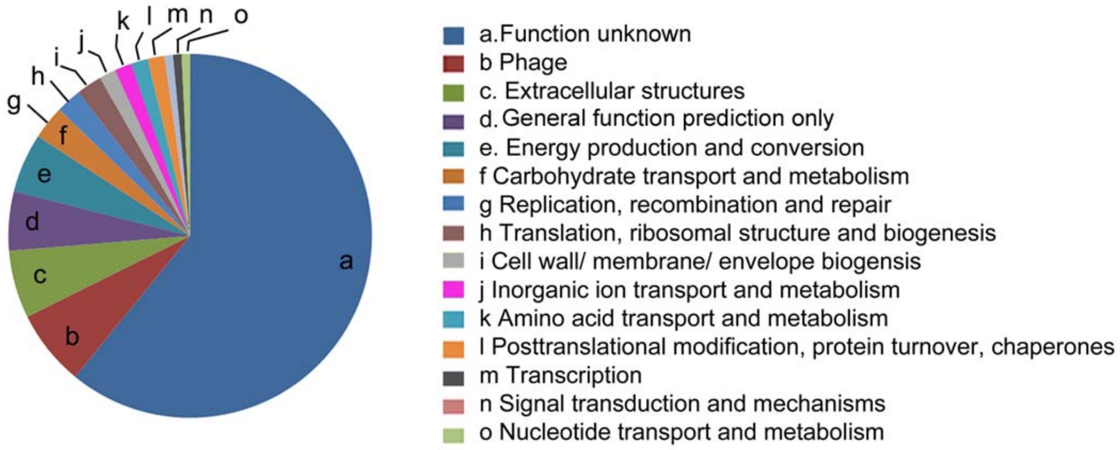

Total genomic comparison of Hif KR494 with *H. influenzae* reference strains, *H. aegyptius, H. haemolyticus* and *H. parainfluenzae.* Unique genes (133 CDSs) of Hif KR494 that consistently lacked homology with any of the aligned species were delineated based on COG database. In particular, after the genes of unknown function, most of the universal unique CDSs of Hif KR494 were phage-related products, followed by extracellular structures. The data represent the universal gene feature of Hif KR494.
